# Expression Pattern of Entire Cytochrome P450 Genes and Response of Defenses in a Metabolic-Herbicide-Resistant Biotype of *Polypogon fugax*

**DOI:** 10.3389/fpls.2022.868807

**Published:** 2022-03-25

**Authors:** Jiajia Yang, Minghao Jiang, Siwei Jia, Min Liao, Haiqun Cao, Ning Zhao

**Affiliations:** ^1^Anhui Province Key Laboratory of Integrated Pest Management on Crops, School of Plant Protection, Anhui Agricultural University, Hefei, China; ^2^Anhui Province Engineering Laboratory for Green Pesticide Development and Application, School of Plant Protection, Anhui Agricultural University, Hefei, China; ^3^School of Agronomy, Anhui Agricultural University, Hefei, China

**Keywords:** fenoxaprop-*P*-ethyl metabolism, P450 gene expression, internal control, mesosulfuron-methyl, *Polypogon fugax*, RT-qPCR

## Abstract

Enhanced herbicide metabolism mediated by cytochrome P450s has been proposed as one of the major mechanisms of resistance to fenoxaprop-*P*-ethyl in a metabolic-herbicide-resistant biotype of Asia minor bluegrass (*Polypogon fugax* Nees ex Steud.). Upon pre-treatment with the P450 inhibitor piperonyl butoxide, a remarkable reduction in metabolic rates of the phytotoxic fenoxaprop-*P* has been observed in the resistant plants, implying that constitutive and/or fenoxaprop-*P*-ethyl-induced up-regulation of specific P450 isoforms are involved in the fenoxaprop-*P*-ethyl resistance. However, which P450 gene(s) were responsible for the metabolic resistance is still unknown. In this present study, based on the abundant gene resources of *P. fugax* established previously, a total of 48 putative P450 genes were isolated from the metabolic-herbicide-resistant plants and used for gene expression analysis. The most suitable reference genes for accurate normalization of real-time quantitative PCR data were first identified in *P. fugax* and recognized as actin (*ACT*), 18S rRNA (*18S*), and ribulose-1,5-bisphosphate carboxylase oxygenase (*RUBP*) under fenoxaprop-*P*-ethyl stress, glyceraldehyde-3-phosphate dehydrogenase (*GAPDH*) and elongation factor 1α (*EF1α*) under mesosulfuron-methyl stress, and *ACT*, *EF1α*, eukaryotic initiation factor 4a (*EIF4A*), and 25S rRNA (*25S*) at different growth stages. Expression analysis of the putative P450 genes revealed that six genes, respectively, annotated as *CYP709B1*, *CYP71A1-4*, *CYP711A1*, *CYP78A9*, *P450-11*, and *P450-39* were up-regulated more than 10-fold in the resistant plants by fenoxaprop-*P*-ethyl treatment, and all of them exhibited constitutively and/or herbicide-induced higher transcript levels in the fenoxaprop-*P*-ethyl-resistant than in the susceptible plants. Three genes, respectively, annotated as *CYPRO4*, *CYP313A4*, and *CYP51H11* constantly up-regulated in the resistant than in the susceptible plants after fenoxaprop-*P*-ethyl treatment. Up-regulated expressions of these specific P450 genes were consistent with the higher P450 contents determined in the resistant plants. These results will help to elucidate the mechanisms for P450-mediated metabolic-herbicide resistance in *P. fugax* as well as other grass weed species.

## Introduction

Asia minor bluegrass (*Polypogon fugax* Nees ex Steud.) is an annual or biennial hexaploid grass species widely spreading in Asia, Eurasia, and North America (Wang et al., 2016; Akopian and Ghukasyan, 2018). In China, *P. fugax* severely infests winter wheat (*Triticum aestivum* L.) fields with a rice (*Oryza sativa* L.) or maize (*Zea mays* L.) rotation. It competes with crop seedlings for natural resources like light, water, and fertilizer, reducing grain yields by up to 40% (Tang et al., 2015). Since the 1990s, the acetyl-CoA carboxylase (ACCase, EC. 6.4.1.2)-inhibiting herbicide fenoxaprop-*P*-ethyl has been widely adopted for control of this noxious weed. However, repeated use has facilitated the development of herbicide resistance in *P. fugax*. Tang et al. (2014) reported the first case of *P. fugax* resistance to fenoxaprop-*P*-ethyl from Sichuan Province, China, and more herbicide resistance of this weed have been in recent years (Zhao et al., 2019; Chen et al., 2020; Yu et al., 2021).

Weed resistance to herbicides is mainly categorized into target-site based resistance (TSR) and non-TSR (NTSR; Gaines et al., 2020). In the last decades, most of the herbicide-resistance cases reported have occurred *via* the former mechanism. TSR is certainly the most studied aspect to date and generally caused by structural changes to the herbicide-binding sites or overproduction of target proteins (Gaines et al., 2019). In contrast, NTSR contains many other mechanisms that reduce the amount of active herbicide reaching the target sites and is a more complex trait. Of these, enhanced herbicide metabolism is a frequently reported NTSR mechanism which under complex genetic controls. There is evidence that enhanced metabolism often involves multiple genes from different herbicide detoxifying-enzyme families, such as cytochrome P450 monooxygenases (P450s), glutathione S-transferases, UDP-glycosyltransferases, and transporter proteins (Délye et al., 2013). Most of the weed plants carrying metabolism-based resistance exhibit multiple-herbicide resistance and pose a great threat to modern agricultural production.

Of the herbicide detoxifying-enzyme families, P450s are ubiquitous hemethiolate monooxygenases that present in the genomes of virtually all organisms (Werck-Reichhart and Feyereisen, 2000). They can catalyze a wide variety of reactions involves monooxygenation and hydroxylation. In higher plants, hundreds of P450 genes exist in their genomes. Generally, the rates of herbicide metabolism are different among plant species, and the selectivity of many herbicides is based on the difference in rates of herbicide metabolism between crop and weed plants (Owen, 1991). In recent years, more and more weed plants exhibit high herbicide-detoxifying P450 activity, suggesting the development of P450-mediated herbicide resistance (Jugulam and Shyam, 2019). Omics-based tools have thus been widely adopted to identify candidate genes related to herbicide detoxification (Giacomini et al., 2017), and some P450 genes from crop and weed species have been demonstrated to metabolize numerous herbicides from various chemical backbones (Iwakami et al., 2019; Kato et al., 2020; Han et al., 2021). Nevertheless, herbicide-detoxifying P450s remain to be identified in most weed species, as many factors such as herbicide application history and genetic background may impact the evolution of P450-mediated herbicide resistance.

Plant P450 gene sequences are rich in diversity and lack of conserved regions. Polymerase chain reaction (PCR) using PERF/W and PFG motifs is a conventional method for isolating putative plant P450 genes, but the low efficiency and the few conserved P450 motifs have limited its application (Iwakami et al., 2014). Transcriptomic is a robust tool to identify unknown genes related to a quantitative trait in specific species without a complete genome. Especially, third-generation sequencing by PacBio platform can provide long-reads up to 10 kb, making it possible to accurately reconstruct full-length splice variants. Therefore, the full-length reference transcriptome assembled by third-generation sequencing will present a whole landscape of genes annotated as related to P450s in a given species. This approach can improve the efficiency of gene amplification and isolate more diverse P450 genes, which will help to investigate the herbicide-detoxifying P450s in arable weed species.

Real-time quantitative PCR (RT-qPCR) is a powerful tool for measuring gene expression and will be essential for deciphering molecular mechanisms of herbicide resistance. More and more studies have suggested that the expression of reference genes may vary among species and under various conditions (Xu et al., 2017). Therefore, it is necessary to systematically identify stably expressed genes as internal controls before conducting any meaningful RT-qPCR analysis. So far, suitable reference genes have been identified in many important crop species (Jarošová and Kundu, 2010; Kong et al., 2016; Kiarash et al., 2018), and lack of detailed genomic information has limited the assessment of reference genes in weed species. Identification of stably expressed gene(s) in these plants under different conditions will provide useful guidelines for accurate gene expression studies.

Previously, we have identified a fenoxaprop-*P*-ethyl-resistant *P. fugax* population (AHHY) and preliminarily characterized its TSR and NTSR mechanisms (Zhao et al., 2019). Trp-1999-Ser mutation of *ACCase* and P450s-mediated enhanced metabolism were simultaneously involved in the resistance phenotypes (Zhao et al., 2019, 2022b). However, whether *ACCase* overexpression played a role in the resistance or not and which P450 gene(s) were responsible for the metabolic resistance are still unknown. Although previous RNA sequencing has suggested several genes may be involved in the resistance (Zhao et al., 2022b), some key genes may be ignored due to sampling simplicity and higher false positive. To address these issues, in the current study, seven candidate reference genes were first isolated from *P. fugax*, and their expression stability was systematically assessed in *P. fugax* plants under herbicide stress as well as other experimental conditions. *ACCase* expression was then determined in different biotypes of *P. fugax* to rule out the gene overexpression as a resistance mechanism. Thereafter, the entire P450 genes were isolated from the full-length transcriptome assembled previously, and their temporal expression patterns were detailedly characterized in *P. fugax* under fenoxaprop-*P*-ethyl stress by RT-qPCR. The comparison of P450 genes between susceptible and fenoxaprop-*P*-ethyl-resistant plants will help to uncover the mechanism for P450-mediated herbicide resistance in *P. fugax*.

## Materials and Methods

### Plant Materials and Growth Condition

Two populations of *P. fugax*—a susceptible population (S, SDTS) and a resistant population (R, AHHY)—were used in this study. Seed collection and herbicide resistance testing for each population have been described in Zhao et al. (2019). Of these, the SDTS was susceptible to the ACCase inhibitor fenoxaprop-*P*-ethyl, while the AHHY had evolved both TSR and NTSR to it. Besides, both populations were susceptible to mesosulfuron-methyl, isoproturon, and cypyrafluone.

Seeds were randomly selected from each population and pre-germinated in Petri dishes containing 5 ml of distilled water. After their germination, 12 seeds were sown into 20-cm-diameter plastic pots filled with loam soil and grown in a controlled greenhouse (25/15°C day/night, 12-h photoperiod, natural light, with ∼75% relative humidity). All the pots were watered and re-arranged every other day.

### Plant Sample Preparation for Expression Stability Analysis

#### Sample Collection at Different Times After Herbicide Treatment

*Polypogon fugax* seedlings at the three- to four-leaves stage were treated with fenoxaprop-*P*-ethyl (69 g L^−1^ emulsion in water, Bayer, Hangzhou, China) at 62.1 g ai ha^−1^ or mesosulfuron-methyl (30 g L^−1^ oil-miscible flowable, Bayer) at 9.0 g ai ha^−1^. Herbicides were sprayed by using a laboratory cabinet sprayer equipped with a Teejet 9503EVS flatfan nozzle (Zhao et al., 2018). A time-course experiment was performed to determine the stably expressed genes in susceptible and resistant plants with or without herbicide treatment. Fresh leaves from each biotype were separately harvested at 0 (untreated), 2, 6, 24, 48, 72, and 96 h after treatment (HAT), after which they were immediately frozen in liquid nitrogen and stored at −80°C until RNA extraction. A total of 56 samples (two populations × two treatments × seven different sampling time points × two biological replicates) were collected in this study. They were divided into two sample subsets based on the herbicide received (i.e., a fenoxaprop-*P*-ethyl subset and a mesosulfuron-methyl subset).

#### Sample Collection at Different Growth Stages

Fresh leaf tissues were separately harvested from the S and R biotypes of *P. fugax* at the three- to four-leaf stage, tillering stage, jointing stage, and heading stage. All samples were immediately frozen in liquid nitrogen and stored at −80°C. A total of 16 samples (two populations × four growth stages × two biological replicates) were collected and grouped into a growth-stage subset.

#### Sample Collection of Different Plant Organs

Fresh roots, stems, leaves, and spikes were separately harvested from the S and R biotypes of *P. fugax* prior to ripening at the fruit-bearing stage. All samples were immediately frozen in liquid nitrogen and stored at −80°C. A total of 16 samples (two populations × four organs × two biological replicates) were collected and grouped into an organ subset.

### Total RNA Extraction and Primary cDNA Synthesis

Total RNA was isolated from each sample using the TRIzol Reagent (Invitrogen, Carlsbad, CA, United States) according to the manufacturer’s procedures. The quality and integrity of the RNA samples were assessed by using a NanoDrop 2000 spectrophotometer (Thermo Fisher Scientific, Middlesex, MA, United States) and by 1% (*w*/*v*) agarose gel electrophoretic analysis, respectively. All the RNA samples used in the following experiments had passed the quality controls. The trace of contaminating genomic DNA (gDNA) was eliminated and the first-strand cDNA was synthesized from approximately 1 μg of total RNA by using the *EasyScript*® All-in-One First-Strand cDNA Synthesis SuperMix for qPCR (One-Step gDNA Removal; TransGen Biotech, Beijing, China) according to the manufacturer’s procedures. The obtained cDNAs were stored at −20°C until further use.

### Candidate Reference Genes Selection and Primer Design

A total of seven candidate genes were selected for the analysis in this study, including actin (*ACT*), eukaryotic initiation factor 4a (*EIF4A*), elongation factor 1α (*EF1*α), 18S rRNA (*18S*), 25S rRNA (*25S*), ribulose-1,5-bisphosphate carboxylase oxygenase (*RUBP*), and glyceraldehyde-3-phosphate dehydrogenase (*GAPDH*; Table 1). These genes are frequently used as internal controls in studies investigating plant responses to various abiotic stresses (Nicot et al., 2005; Dombrowski and Martin, 2009; Duhoux and Délye, 2013; Wrzesińska et al., 2016; Kiarash et al., 2018). Sequence for each gene was obtained by querying the full-length transcriptome of *P. fugax* assembled recently (Accession number: MZ221767–MZ221773). Primers were designed using Primer Premier v.5.0 software (PREMIER Biosoft International, Palo Alto, CA, United States) and were synthesized by TsingKe Biotech Co., Ltd. (Beijing, China).

**Table 1:** Basic characteristics of seven candidate reference genes for RT-qPCR.

Gene^a^	GenBank accession	Primer sequences (5′–3′)	*T*_m_^b^ (°C)	Amplicon length (bp)	Amplification efficiency (%)	Correlation coefficient	Average Cq value
*ACT*	MZ221767	AGGACGAGTACGACGAATCTG	60	221	87.6	0.962	25.5
		CGTACTTGCACTTGCACATGG					
*EIF4A*	MZ221768	TCCGTCGGGAACCAATATAAGC	60	124	97.7	0.980	26.4
		AGCGAATCCGTGGCCCGGATTCGAT					
*EF1α*	MZ221769	CAGATCGGCAACGGCTACGC	60	277	92.9	0.979	22.4
		CCTTCTCCACGCTCTTGATGACAC					
*18S*	MZ221770	GTTCTTAGTTGGTGGAGCG	56	124	103.6	0.962	12.6
		CTAAACGGCGATAGTCCC					
*25S*	MZ221771	CCACTGTCCCTGTCTACTATCC	56	140	96.4	0.995	13.5
		CTCCCACTTATCCTACACCTCT					
*RUBP*	MZ221772	TGCCAGCTCTGACCGAAATCTTTG	60	211	102.9	0.983	16.1
		GCGGCTAGTTCAGGACTCCATTTG					
*GAPDH*	MZ221773	CCACTAACTGCCTTGCTCCTCTTG	60	262	91.7	0.992	24.8
		ACATCAACGGTTGGAACACGGAAG					

a ACT, actin; EIF4A, eukaryotic initiation factor 4a; EF1*α, elongation factor 1α*; 18S, 18S rRNA; 25S, 25S rRNA; RUBP, ribulose-1,5-bisphosphate carboxylase oxygenase; GAPDH, glyceraldehyde-3-phosphate dehydrogenase.

b *T*_m_, melting temperature.

### PCR Reaction and Verification of Amplified Products

Reverse transcription (RT)-PCR was performed to amplify specific fragment of each candidate gene from cDNA using 2 × Taq MasterMix (Vazyme, Nanjing, China). PCR product sizes were checked on 1% (*w*/*v*) agarose gels, and the expected amplicons were sequenced to confirm the amplification of target genes (Supplementary Table S1).

RT-qPCR was performed in 0.2 ml 8-Tube qPCR Strips (PCR-0108-LP-RT-C, Union City, CA, United States) using CFX96 Touch Real-Time PCR Detection System (Bio-Rad, Hercules, CA, United States). PCR reactions were prepared by using 2 × TransStart Top Green qPCR SuperMix (TransGen Biotech) as described elsewhere (Zhao et al., 2018). Two-step RT-qPCR was adopted for primers whose melting temperature (*T*_m_) was ≥ 60°C, and three-step RT-qPCR was adopted for the other primers. Each PCR reaction was performed in technical triplicates with non-template controls (NTCs) and no reverse transcriptase (no-RT) controls.

PCR product specificity of each primer pair was verified through melting curve analyses, and the amplification efficiency (*E*) and correlation coefficients (*r*^2^) of each primer pair were calculated using the standard curve method (Zhao et al., 2018).

### Reference Gene Expression Stability Analysis

Three statistical algorithms, including geNorm (Vandesompele et al., 2002), NormFinder (Andersen et al., 2004), and the comparative ΔCq method (Silver et al., 2006) were used to systematically assess the expression stability of each candidate gene. A web-tool RefFinder^1^ was finally used to compare the results of different statistical programs to generate a comprehensive ranking of stability for the candidate genes (Xie et al., 2012). In the comparative ΔCq method and RefFinder, the raw Cq values calculated by CFX maestro v.4.1 software (Bio-Rad) were directly used. In geNorm and NormFinder, the raw Cq values were transformed to relative quantities by the 2^−ΔCq^ method, with the lowest Cq value serving as a calibrator.

### *ACCase* Gene Expression Analysis

Based on the stably expressed reference genes identified, *ACCase* gene expression was compared on extracts from three S (SDTS) and three R (AHHY) plants with each sample subjected to a RT-qPCR analysis in triplicate. The primer pair ACCase-F1 (5′-CACTGAAGGGAAGCGATTGGGT-3′) and ACCase-R1 (5′-CTTCAGATGCATTTGTTTGAG-3′) with a T_m_ of 60°C was designed to amplify a 175-bp fragment of *ACCase*. Because the different alleles of *ACCase* have a very high homology (Zhao et al., 2019), the *ACCase* expression determined here represents the total expression of all *ACCase* alleles. The oligonucleotide was designed to overlap to intron junction by analysis of the assembled transcriptome to the corresponding genomic region of maize (GenBank No. XM_008664833) using EMBOSS EST2Genome (Rice et al., 2000). RT-qPCR was performed using the two-step method as described above, and the results were analyzed by the 2^−ΔΔCq^ method using CFX Maestro v.4.1 software (Bio-Rad). When comparing the expression levels of a target gene between two groups of samples, the software considers the respective amplification efficiencies of target gene and reference gene(s).

### P450 Content Assay

P450 contents for the S and R biotypes were determined through the method described by Wang et al. (2013) with minor modifications. Seeds of the S and R biotypes were pre-germinated and sown in square plastic pots (10 cm × 10 cm × 8.5 cm) filled with quartz sand. After planting, each pot was put in a plastic bowl containing half-strength Hoagland’s nutrient solution (Coolaber, Beijing, China), wrapped with aluminum foil to exclude light, and grown in a climatic chamber at 25/15°C without light. The nutrient solution was changed every 3 days. At 18 days after planting, the seedlings were sprayed with fenoxaprop-*P*-ethyl at 31.1 g ai ha^−1^ as described above. The etiolated shoots of the weed seedlings were then collected at 0, 1, 2, 3, 5, and 7 days after treatment and used for microsome isolation. P450 content in each sample was quantified using reduced carbon monoxide difference spectroscopy and expressed as nmol mg^−1^ microsomal protein (Estabrook and Werringloer, 1978; Jefcoate, 1978). The experiment was conducted twice with each treatment had three replicates.

### Isolation of Putative P450 Genes

In our previous study, a reference transcriptome containing 24,972 full-length isoforms with a N50 of 2,575 bp was established for *P. fugax* through third-generation PacBio sequencing (Zhao et al., 2022b). Gene functional annotation by BLAST against the NCBI non-redundant protein (Nr) database revealed that a total of 169 unigenes were annotated as related to putative P450s (Supplementary Table S2). However, *P. fugax* is a hexaploid (2*n* = 6*x* = 42) grass weed, and multiple alleles encoding a specific enzyme were expected to be present in this polyploid species (Akopian and Ghukasyan, 2018; Zhao et al., 2019). Therefore, in the current study, the contigs having a high sequence similarity (≥80%) and sharing a same functional annotation were considered as the different alleles encoding a same P450 enzyme (Supplementary Table S2). Based on the selection standard, a total of 48 putative P450 genes were successfully identified and isolated from the reference transcriptome (Supplementary Table S3).

### Expression Analysis of Putative P450s by RT-qPCR

Plants of the S and R lines were grown together in a growth chamber at 25/15°C day/night with a 12-h photoperiod (200 μmol m^−2^ s^−1^ photosynthetic photon flux density, with ~75% relative humidity). Weed seedlings at the three- to four-leaves stage were treated with fenoxaprop-*P*-ethyl at the field-recommended rate (62.1 g ai ha^−1^). Fresh shoots of the S and R plants were sampled at the same time at 0 (untreated), 6, 12, and 24 HAT, frozen in liquid N_2_, and stored at −80°C. RNA was extracted from each sample using the TRIzol Reagent (Invitrogen) and assessed as described above.

Primers were designed using Primer Premier v.5.0 software (PREMIER Biosoft International) and summarized in Supplementary Table S3. For the genes encoded by multiple alleles, primers were designed based on the conserved regions of different alleles. RT-qPCR was performed on the CFX96 Touch Real-Time PCR Detection System (Bio-Rad). cDNA was prepared as described above from 0, 6, 12, and 24-h RNAs extracted from the S and R plants. The expression of each putative P450 gene was normalized relative to the mean of *ACT*, *18S*, and *RUBP*, and the results were analyzed by the 2^−ΔΔCq^ method. Two threshold values including the fold change (twofold) and *t*-test (*p* < 0.05) were used to determine the up- or down-regulation of a target gene by fenoxaprop-*P*-ethyl treatment.

## Results

### Characterization of the Candidate Reference Genes

A total of seven candidate genes were selected and assessed in this study (Table 1). Every gene showed a single DNA band in agarose gel electrophoresis (Supplementary Figure S1) and a single-peak melting curve in RT-qPCR analysis (Supplementary Figure S2), confirming the absence of primer dimers and non-specific amplicons. Gene sequencing and alignment suggested the specific amplification of the targeted amplicons and the absence of gDNA contaminations (Supplementary Table S1). The NTCs and no-RT controls detected no amplicon peak or RT-qPCR signal, indicating the absence of cross-contamination. The RT-qPCR efficiencies (*E*) obtained for each primer pair ranged from 85 to 105%, and the corresponding correlation coefficients (*r*^2^) comprised between 0.95 and 1.00 (Table 1; Supplementary Figure S3). This indicates the developed RT-qPCR system was reliable and comparable.

### Expression Profiles of Seven Candidate Reference Genes

**Figure 1:**
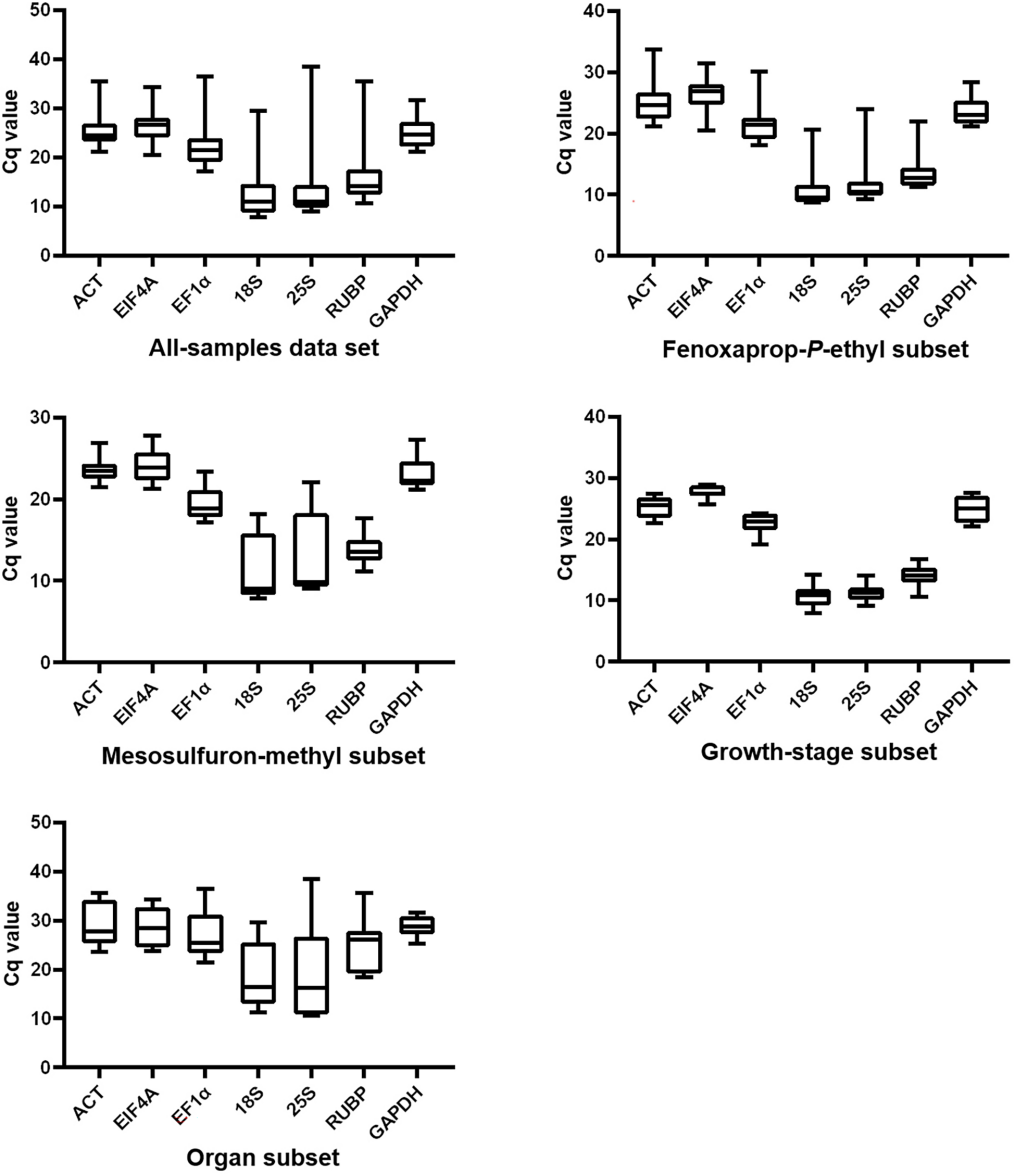
Overview of transcript levels of seven candidate reference genes in *Polypogon fugax* under herbicide stress as well as other experimental conditions. Top and bottom borders represent the 25th and 75th percentiles, respectively. The horizontal line within the box indicates the median. Whiskers represent the maximum and minimum values.

The Cq values of each gene generated in RT-qPCR were used to create box-and-whisker plots to present an overview of the transcript levels under different experimental conditions (Figure 1). In this study, different genes showed distinct expression levels, with mean Cq values ranging from 12.6 to 26.4 (Table 1). Each specific gene exhibited a similar expression pattern in *P. fugax* plants under different experimental conditions (Figure 1). Based on the median and mean Cq values, *18S* and *EIF4A* were considered to express to higher and lower levels than the other six genes, respectively.

### Determination of the Most Stable Genes in Different Sample (Sub)sets

*Polypogon fugax* samples were classed into different data (sub)sets according to their corresponding experimental conditions: an all-samples data set, a fenoxaprop-*P*-ethyl subset, a mesosulfuron-methyl subset, a growth-stage subset, and an organ subset. The expression stability of the seven candidate genes was ranked in each sample grouping.

In the fenoxaprop-*P*-ethyl subset, geNorm indicated *18S*, *25S, RUBP*, *ACT*, and *EF1α* as the stable genes (Supplementary Figure S4) and suggested three genes would beadequate for accurate data normalization (Supplementary Figure S5). NormFinder identified *ACT* as the most stable gene and suggested *EF1α* and *18S* should be conjointly used for accurate data normalization (Supplementary Table S4). The comparative ΔCq method ranked *ACT*, *18S*, *EF1α*, and *RUBP* as the four most stable genes (Supplementary Table S5). Considering the comprehensive ranking of stability presented by RefFinder (Table 2), we recommended *ACT*, *18S*, and *RUBP* as suitable reference genes in *P. fugax* leaves treated with fenoxaprop-*P*-ethyl.

**Table 2:** Ranking of seven candidate reference genes according to their expression stability using RefFinder.

Rank	All-samples set		Fenoxaprop-*P*-ethyl subset		Mesosulfuron-methyl subset		Growth stage subset		Organ subset	
	Gene	GM	Gene	GM	Gene	GM	Gene	GM	Gene	GM
1	*EF1α*	1.41	*ACT*	2.30	*GAPDH*	1.41	*25S*	1.78	*RUBP*	1.97
2	*GAPDH*	1.73	*18S*	2.34	*EF1α*	2.21	*EF1α*	2.21	*EF1α*	2.00
3	*18S*	2.99	*RUBP*	3.13	*ACT*	2.28	*EIF4A*	2.63	*18S*	2.91
4	*EIF4A*	3.50	*EF1α*	3.31	*RUBP*	2.99	*ACT*	3.50	*EIF4A*	3.13
5	*RUBP*	4.68	*25S*	3.50	*EIF4A*	4.73	*GAPDH*	3.60	*GAPDH*	3.34
6	*ACT*	5.05	*GAPDH*	4.30	*18S*	6.00	*RUBP*	5.66	*ACT*	5.66
7	*25S*	7.00	*EIF4A*	5.05	*25S*	7.00	*18S*	5.73	*25S*	6.24

GM, geometric mean of weights.

In the mesosulfuron-methyl subset, different algorithms produced distinct results (Supplementary Figure S4, Supplementary Tables S4, S5). geNorm indicated that two genes would be adequate for accurate data normalization (Supplementary Figure S5). Based on the comprehensive ranking of stability calculated by RefFinder (Table 2), we considered the *GAPDH* and *EF1α* as suitable internal controls.

Similarly, *ACT*, *EF1α*, *EIF4A*, and *25S* were recommended as a suitable gene combination for data normalization at different growth stages of *P. fugax* (Table 2). However, no specific gene or gene combination was suitable for accurate data normalization in the different organs of *P. fugax*, nor in the all-samples data set.

### *ACCase* Expression in *Polypogon fugax* With or Without Fenoxaprop-*P*-ethyl Treatment

**Figure 2:**
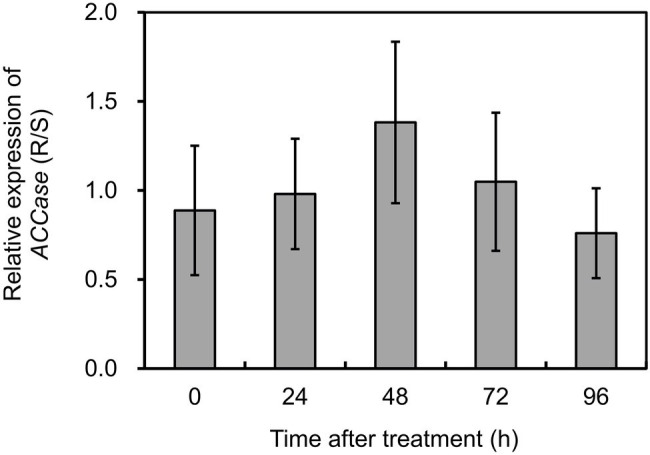
Relative expression levels of *ACCase* in the R relative to the S plants of *Polypogon fugax* at 0 (control), 24, 48, 72, and 96 h after fenoxaprop-*P*-ethyl treatment. Data are the mean values ± SEM.

To investigate if target gene overexpression participated in the resistance phenotypes, the transcript abundance of *ACCase* was compared in the S and R biotypes of *P. fugax*. The expression of *ACCase* was normalized relative to the mean of *ACT*, *18S*, and *RUBP*. PCR efficiency (*E*) for *ACCase* was 98.6% with a *r*^2^ of 0.969, and the mean Cq value was 25.17. Based on the data obtained, *ACCase* expression showed no significant difference between plants with contrasting phenotypes with or without herbicide treatment (Figure 2). This indicates that *ACCase* overexpression did not occur in the R biotype of *P. fugax*.

### P450 Content in Different Biotypes of *Polypogon fugax*

**Figure 3:**
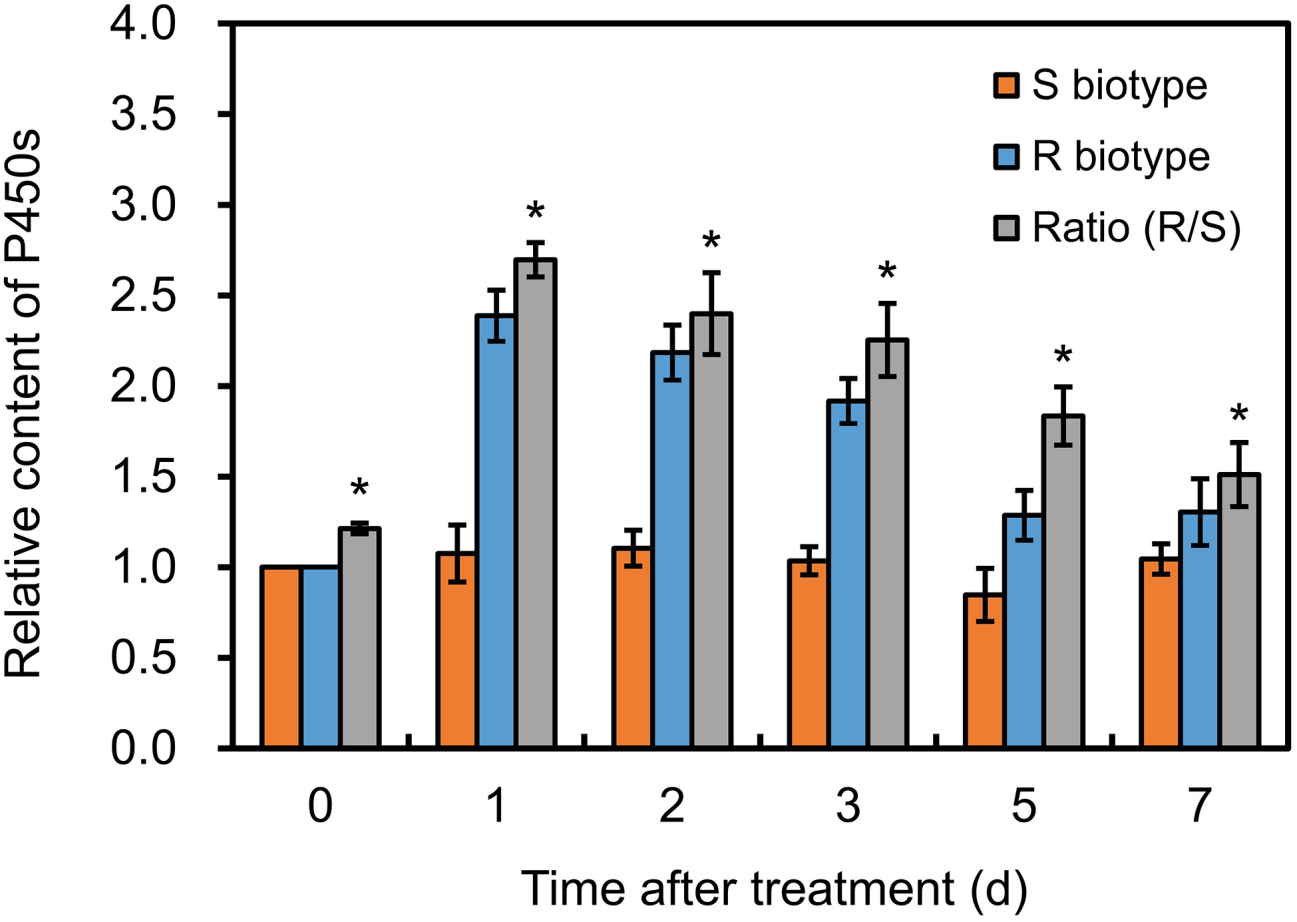
Changes in cytochrome P450 contents in the S and R biotypes of *Polypogon fugax* after fenoxaprop-*P*-ethyl treatment. The difference in P450 contents was presented as ratio of the R relative to the S biotype. Data are the mean values ± SEM. *p < 0.05.

Before herbicide treatment, P450 content in the R plants was approximately 0.76 nmol mg^−1^ protein, which was significantly higher (*p* < 0.05) than that in the S plants (Figure 3). After fenoxaprop-*P*-ethyl treatment, the P450 content increased markedly by 2.39-fold in the R plants, while no serious change in the S plants (Figure 3). Notably, the P450 content in the R plants was always higher (*p* < 0.05) than that in the S plants during 1–7 days after fenoxaprop-*P*-ethyl treatment (Figure 3). This suggested that P450s could be induced by fenoxaprop-*P*-ethyl treatment and were expected to be involved in the metabolism-based resistance phenotypes.

### Gene Expression Patterns of P450s Under Fenoxaprop-*P*-ethyl Stress Condition

**Figure 4:**
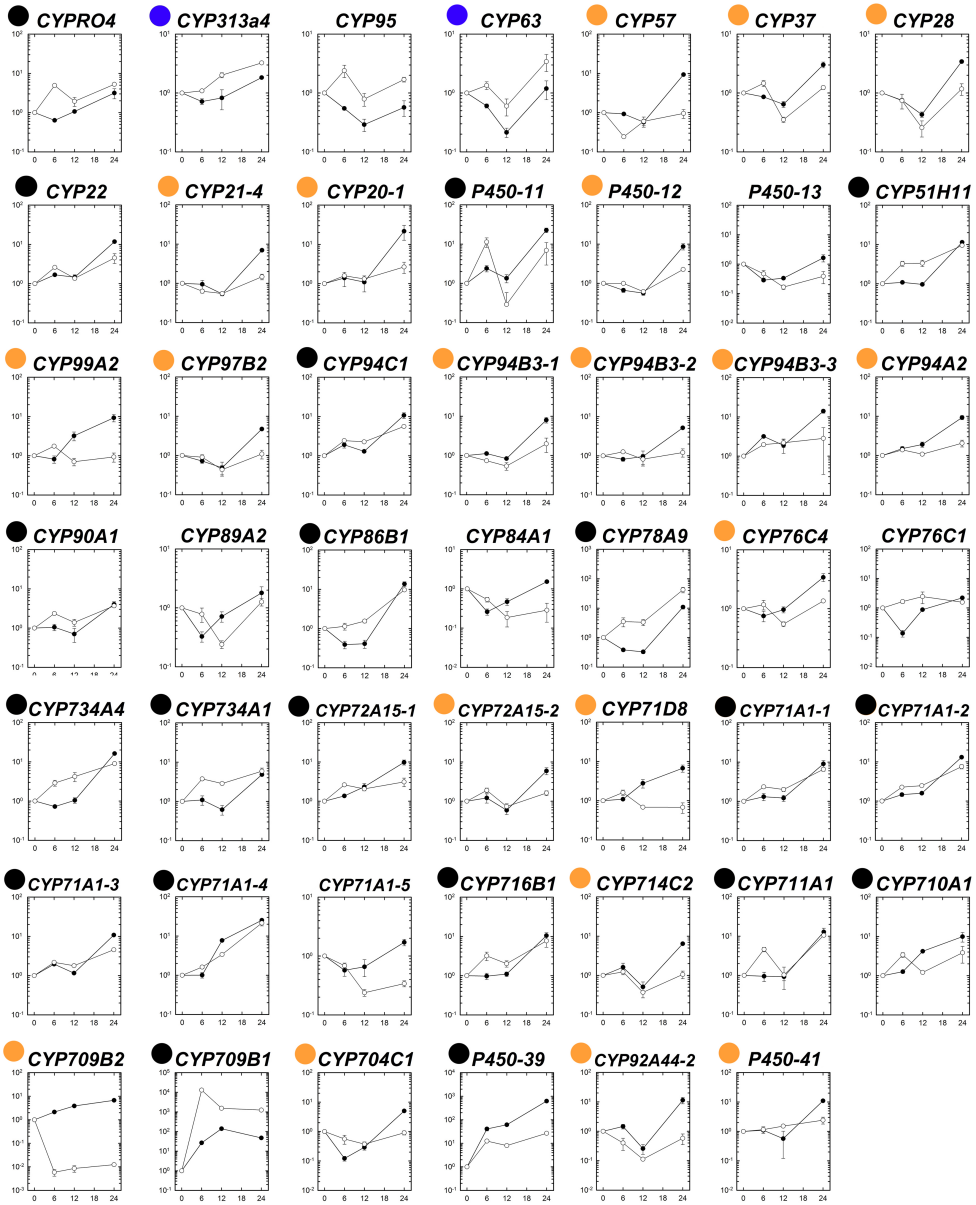
Temporal dynamic changes in relative transcript levels of each putative P450 gene in the S (●) and R (○) lines of *Polypogon fugax* under fenoxaprop-*P*-ethyl stress. The X-axis represents the time (h) after fenoxaprop-*P*-ethyl treatment, and the *Y*-axis represents the transcript levels relative to the untreated sample (0 h). Data are the mean values ± SEM. Black dots marked genes were up-regulated more than threefold in both the S and R lines by fenoxaprop-*P*-ethyl treatment for at least one time point, orange dots marked genes were up-regulated more than threefold only in the S line, and blue dots marked genes were up-regulated more than threefold only in the R line.

The expression patterns of the 48 putative P450 genes were first analyzed in the susceptible and fenoxaprop-*P*-ethyl-resistant biotypes at different times after herbicide treatment. Generally, these P450 genes performed differently in their relative expression levels during 0 to 24 HAT (Figure 4). Of these, 22 genes were up-regulated more than threefold for at least one time point after fenoxaprop-*P*-ethyl treatment in the R line (black and blue dots marked genes in Figure 4), 40 genes were up-regulated more than threefold in the S line (black and orange dots marked genes in Figure 4), and 20 genes were up-regulated more than threefold in both the S and R lines (black dots marked genes in Figure 4). The expression levels of the herbicide-treatment-inducible genes also differed substantially. In the R line, six genes, respectively, annotated as *CYP709B1*, *CYP71A1-4*, *CYP711A1*, *CYP78A9*, *P450-11*, and *P450-39* were up-regulated more than 10-fold, and the strongest up-regulation was observed for the gene annotated as *CYP709B1* which had a more than 10,000-fold increase in expression at 6 h. In comparison, 18 genes were up-regulated more than 10-fold in the S line, and the strongest up-regulation was observed for the gene annotated as *P450-39* (more than 600-fold).

### Comparison of Gene Expression Between Fenoxaprop-*P*-ethyl-Resistant and Susceptible Lines of *Polypogon fugax*

**Figure 5:**
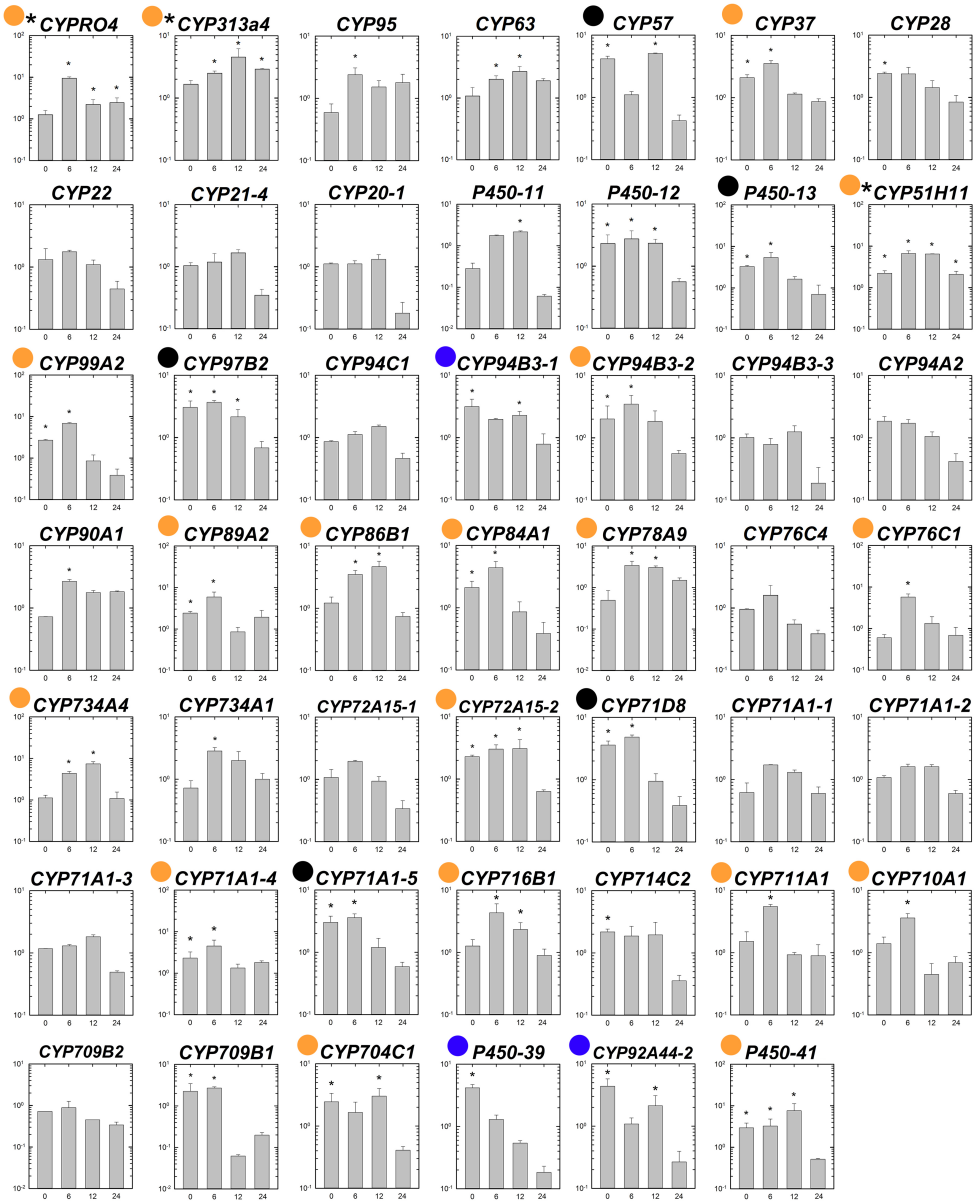
Comparison of relative expression levels of each putative P450 gene in the S and R lines of *Polypogon fugax* before and after fenoxaprop-*P*-ethyl treatment. The *X*-axis represents the time (h) after fenoxaprop-*P*-ethyl treatment, and the *Y*-axis represents the ratio of relative transcript levels in the R line relative to that in the S line at each sampling time point. Data are the mean values ± SEM. Blue dots marked genes were constitutively up-regulated more than threefold in the R than in the S line, orange dots marked genes were fenoxaprop-*P*-ethyl-induced up-regulated more than threefold in the R than in the S line for at least one time point, and black dots marked genes were both constitutively and herbicide-induced up-regulated more than threefold in the R than in the S line. Asterisk marked genes were constantly up-regulated in the R than in the S line during 6–24 h after treatment. ^*^Foldchange ≥2 and *p* < 0.05.

The transcript levels of the 48 putative P450 genes were compared between the susceptible and fenoxaprop-*P*-ethyl-resistant biotypes at 0 (control), 12, 24, and 48 h after herbicide treatment. Under non-herbicide-stressed conditions, eight genes were constitutively up-regulated more than threefold in the R relative to the S line (black and blue dots marked genes in Figure 5). These include one CYP57 gene, two CYP71 genes, one CYP92 gene, one CYP94 gene, one CYP97 gene, and two other genes (*P450-13* and *P450-39*) without specific annotations. All the above genes showed moderate transcription increases ranging from threefold to fivefold. Under fenoxaprop-*P*-ethyl stress, 24 genes were up-regulated more than threefold in the R relative to the S line for at least one time point (black and orange dots marked genes in Figure 5). Of these, three genes had constantly higher transcript abundances in the R than in the S line during 6–24 HAT, including *CYPRO4*, *CYP313A4*, and *CYP51H11* (asterisk marked genes in Figure 5). The maximum difference was observed for *CYPRO4* at 6 HAT, with an approximately 10-fold difference in transcript abundance. Similarly, the transcript levels of *CYP51H11* also showed the maximum difference at 6 HAT. By contrast, the maximum difference in *CYP313A4* transcript levels was observed at 12 HAT, with more than fourfold difference in transcript abundance.

### Selection of Potential Fenoxaprop-*P*-ethyl Metabolism-Related P450 Genes

As described in the above two sections, a total of six genes were up-regulated more than 10-fold in the R line by fenoxaprop-*P*-ethyl treatment. The transcript levels of these six genes were then compared between the susceptible and fenoxaprop-*P*-ethyl-resistant lines. Of these, *CYP709B1*, *CYP71A1-4,* and *P450-39* were constitutively up-regulated in the R relative to the S line, while *CYP709B1*, *CYP71A1-4, CYP711A1*, *CYP78A9*, and *P450-11* were fenoxaprop-*P*-ethyl-induced up-regulated in the R relative to the S line for at least one time point after fenoxaprop-*P*-ethyl treatment.

To improve the rigor of the gene selection and filter possible false-positive genes, we selected the candidate fenoxaprop-*P*-ethyl metabolism-related P450 genes according to the following criteria: the gene (1) was up-regulated by herbicide treatment more than 10-fold in the R line and (2) up-regulated in the R relative to the S line before and/or after fenoxaprop-*P*-ethyl treatment. In addition, the genes constantly up-regulated in the R relative to the S line after herbicide treatment were also selected. Therefore, a total of nine genes separately annotated as *CYPRO4*, *CYP313A4*, *CYP51H11*, *CYP709B1*, *CYP71A1-4, CYP711A1*, *CYP78A9*, *P450-11*, and *P450-39* were identified as potential fenoxaprop-*P*-ethyl metabolism-related genes in *P. fugax*.

## Discussion

Overexpression of genes encoding the target enzymes such as ACCase or herbicide-detoxifying ones such as P450s is a common mechanism of herbicide resistance (Gaines et al., 2020). To investigate the internal molecular mechanisms, gene expression analysis by RT-qPCR is usually conducted in weed plants subjected to herbicide stress. A set of stably expressed reference genes is fundamental for RT-qPCR data normalization in specific plant species. *Polypogon fugax* is one of the most problematic weeds in the world, which severely infests Chinese wheat fields (Tang et al., 2015). Our previous study has found that the *P. fugax* population AHHY was highly resistant to the ACCase-inhibiting herbicide fenoxaprop-*P*-ethyl, and pre-treatment with the P450 inhibitor piperonyl butoxide markedly increased their susceptibility to fenoxaprop-*P*-ethyl (by c. 55%; Zhao et al., 2019). Here we further determined significantly higher P450 contents in the AHHY than in the susceptible plants before and after fenoxaprop-*P*-ethyl treatment (Figure 3), implying that P450s are most likely to play a key role in the resistance. To determine which P450 genes could be responsible for the metabolic resistance, this current study first identified a set of stably expressed reference genes for RT-qPCR data normalization in *P. fugax* under herbicide stress as well as other experimental conditions. The entire P450 genes were then successfully isolated from the full-length transcriptome established previously, and their transcript abundances were compared between susceptible and fenoxaprop-*P*-ethyl-resistant biotypes of *P. fugax* with or without herbicide treatment.

Since the first study identifying *TUB*, *GAPDH*, and *UBQ* as the suitable internal controls in *A. myosuroides*, stably expressed reference genes have also been determined in several other arable weeds subsequently (Xu et al., 2016, 2017; Scarabel et al., 2017; Zhao et al., 2018; Su et al., 2020). In this study, seven candidate reference genes stably expressed in different plant species were selected and assessed by using two *P. fugax* populations with contrasting phenotypes. Three algorithms, namely geNorm, NormFinder, and the comparative ΔCq method, were used to determine the most stably expressed genes. Although the results calculated from different algorithms were similar in each data (sub)set, the specific ranking of expression stability was different. Therefore, RefFinder was finally used to generate an overall ranking based on the geometric mean of weights of each gene calculated by each algorithm. Generally, genes with average Cq values ranged from 15.0 to 30.0 were acceptable for using as internal controls (Picard et al., 2006). In the present study, the expression levels of candidate genes were varied, with their mean Cq values ranged from 12.6 to 26.4. All the seven candidate genes were included as optional genes to determine the most stably expressed genes in *P. fugax*. The most suitable reference genes characterized in the invasive weed *P. fugax* under herbicide stress as well as other experimental conditions will greatly facilitate studies related to biological science in this grass species.

*Polypogon fugax* is reported to be a hexaploid grass species (Akopian and Ghukasyan, 2018), and multiple copies of *ACCase* are expected in this weed. Previously, we had isolated at least four loci encoding plastidic ACCase from resistant *P. fugax* plants and found the Trp-1999-Ser mutation occurred in their *ACCase1,1–2* alleles (Zhao et al., 2019). However, if *ACCase* overexpression was also involved in the resistance remains unclear. In the present study, triplicated experiments consistently revealed similar ΔCq values for *ACCase* between the S and R biotypes, indicating that the R biotype survived the fenoxaprop-*P*-ethyl treatment without involving in the overexpression of the plastid *ACCase*. Similar results are also observed in cyhalofop-butyl-resistant Chinese sprangletop (*Leptochloa chinensis* (L.) Nees; Deng et al., 2019; Zhao et al., 2022a) and mesosulfuron-methyl-resistant American sloughgrass (*Beckmannia syzigachne* Steud.; Wang et al., 2019), in which target-gene overexpression is not involved in the resistance phenotypes.

In recent years, gene duplication of 5-enolpyruvylshikimate-3-phosphate synthase (*EPSPS*) has been frequently identified in glyphosate-resistant biotypes of arable weeds (Gaines et al., 2010; Salas et al., 2012; Nandula et al., 2014; Chatham et al., 2015; Malone et al., 2016; Dillon et al., 2017). This implies there are “easier” evolutionary solutions, such as target-site mutation and enhanced herbicide metabolism, to achieve resistance to the majority of other herbicides (Tranel, 2017). Recently, Laforest et al. (2017) reported *ACCase* amplification (about fivefold to eightfold more copies) as a resistance mechanism to fenoxaprop-*P*-ethyl in large crabgrass (*Digitaria sanguinalis*), indicating the gene amplification may be not a rare herbicide-resistance mechanism. Target gene overexpression was also identified in several other weed species exhibiting resistance to herbicides with different modes of action (Zhao et al., 2018; Sen et al., 2021). Although *ACCase* overexpression is not related to the fenoxaprop-*P*-ethyl resistance in *P. fugax*, relevant reports still inspire us to investigate gene overexpression or amplification as a potential resistance mechanism more routinely.

P450s-medidated enhanced metabolism has been identified as a major NTSR mechanism in many herbicide-resistant weed biotypes, such as Japanese foxtail (*Alopecurus japonicus* Steud.; Chen et al., 2018), tall morningglory (*Ipomoea purpurea* (L.) Roth; Van Etten et al., 2020), corn poppy (*Papaver rhoeas* L.; Torra et al., 2021), and poverty brome (*Bromus sterilis* L.; Sen et al., 2021). As documented, plant P450s catalyze diverse chemical reactions, while the reactions for P450-mediated herbicide metabolism primarily involve alkyl-hydroxylation, *N*-demethylation, *O*-demethylation, and aryl-hydroxylation (Dimaano and Iwakami, 2021). Higher P450 activity may be caused by higher specific activities of the enzymes in the resistant plants and/or by higher expression levels of the genes encoding the P450 enzymes (Iwakami et al., 2014). In the present study, higher P450 contents were also determined in the resistant than in the susceptible plants without or with fenoxaprop-*P*-ethyl treatment. A total of 48 putative P450 genes were then isolated from *P. fugax*, and they showed distinct expression patterns in the S and R plants. Of these, six genes, respectively, annotated as *CYP709B1*, *CYP71A1-4*, *CYP711A1*, *CYP78A9*, *P450-11*, and *P450-39* were fenoxaprop-*P*-ethyl-induced up-regulated more than 10-fold in the R plants, and all of them showed higher transcript levels in the R than in the S plants for at least one sampling time point. Three genes, respectively, annotated as *CYPRO4*, *CYP313A4*, and *CYP51H11* constantly up-regulated in the R than in the S plants after fenoxaprop-*P*-ethyl treatment. Collectively, these results indicate the above nine genes can be regarded as the primary candidates for fenoxaprop-*P*-ethyl metabolism mediated by higher P450 activity. Interestingly, compared with our previous omics analysis (Zhao et al., 2022b), several new P450s except that annotated as into families of CYP709 and CYP81 were additionally identified as major candidates for fenoxaprop-*P*-ethyl metabolism. The temporal dynamic changes in P450 expressions under herbicide stress may fill the gap caused by sampling simplicity and higher false positive in omics analysis and thus further supplement the results. Notably, herbicide selectivity is normally guaranteed by the crop-specific high expression of herbicide-metabolizing gene(s) (Iwakami et al., 2014). It will be of great significant to investigate whether these genes are orthologous genes for crop selectivity, which may reveal if the resistance is conferred by completely different P450 family members.

In summary, here we systematically characterized the expression pattern of entire P450 genes in a metabolic-herbicide-resistant biotype of *P. fugax*. The basal and/or herbicide-induced up-regulation of several specific P450 genes was positively related to the higher P450 contents in the resistant plants, which can be served as a foundation for uncovering the mechanism for P450-mediated herbicide resistance. Future studies such as ectopic overexpression and gene knockdown will aid in elucidating the molecular mechanisms for metabolic-herbicide resistance in *P. fugax*.

## Data Availability Statement

The original contributions presented in the study are included in the article/Supplementary Material, further inquiries can be directed to the corresponding author.

## Author Contributions

NZ designed the research study. JY, MJ, and SJ performed the experiments. NZ and JY carried out data analysis. NZ wrote the manuscript. All authors contributed to the article and approved the submitted version.

## Funding

This research was funded by the Anhui Provincial Natural Science Foundation (No. 2108085QC115), the National Natural Science Foundation of China (No. 32102237), and the Talent Research Project of Anhui Agricultural University (No. rc342004).

## Conflict of Interest

The authors declare that the research was conducted in the absence of any commercial or financial relationships that could be construed as a potential conflict of interest.

## Publisher’s Note

All claims expressed in this article are solely those of the authors and do not necessarily represent those of their affiliated organizations, or those of the publisher, the editors and the reviewers. Any product that may be evaluated in this article, or claim that may be made by its manufacturer, is not guaranteed or endorsed by the publisher.

## Footnotes

^1^ https://www.heartcure.com.au/reffinder/
