# Supplementary material for: Expression Pattern of Entire Cytochrome P450 Genes and Response of Defenses in a Metabolic-Herbicide-Resistant Biotype of Polypogon fugax
Source: Front Plant Sci. 2022 Mar 25;13:868807. doi: 10.3389/fpls.2022.868807 (PMC8990753; doi:10.3389/fpls.2022.868807)
Supplement: Supplementary file 1 [file Data_Sheet_1.PDF]

Supplementary material

Expression pattern of entire cytochrome P450 genes and response of defenses in a metabolic-herbicide-resistant biotype of *Polypogon fugax*

Jiajia Yang[1,2], Minghao Jiang[1,2], Siwei Jia[3], Min Liao[1,2], Haiqun Cao[1,2], Ning Zhao[1,2*]

[1] Anhui Province Key Laboratory of Integrated Pest Management on Crops, School of Plant Protection, Anhui Agricultural University, Hefei 230036, China

[2] Anhui Province Engineering Laboratory for Green Pesticide Development and Application, School of Plant Protection, Anhui Agricultural University, Hefei 230036, China

[3] School of Agronomy, Anhui Agricultural University, Hefei 230036, China

[*] Author for correspondence: Ning Zhao, School of Plant Protection, Anhui Agricultural University, Hefei 230036, Anhui Province, China. E-mail: zhaon@ahau.edu.cn

Table S1. Amplicon sequences of seven candidate genes obtained using RT-PCR.

Gene	Sequences (5’−3’)
ACT	AGGACGAGTACGACGAATCTGGCCCAGCAATCGTCCACAGGAAGTGCTTCTGATCTCT<br>TGCAAGTGCTCCGCCGTTATTGTCTAGTCTTGGAGTCATGTTTGGTTCATTCTTCTAGAT<br>ATGAATTGCAGATTTGCAAGCTACTATGTTTTTTTCCAGACATGAGTACTCTCAGGATAT<br>GCCACTTATATATGTAGTGGCTCCATGTGCAAGTGCAAGTACG
EIF4A	TCCGTCGGGAACCAATATAAGCCGCCTTTCTCTCTCCCCACCTCCACCAAACCCTTGTT<br>CGTCGTCTCCCTCCCCGCACCACCGAAAAGTTCCAATCGATCGAATCCGGGCCACGGA<br>TTCGCT
EF1α	CAGATCGGCAACGGCTACGCCCCAATGCTGGACTGCCACACGTCCCACATCGCTGTCA<br>AGTTCTCTGAGATCCAGACCAAGTTCGACAGGCGTTCTGGCAAGGAGATTGAGGCCTT<br>CCCCAAGTTCCTCAAGAACGGTGATGCTGGTTTCGTGAAGATGATTCCCACCAAGCCC<br>ATGGTGGTGGAGACCTTCGCCCAGTACCCTCCCCTTGGACGCTTTGCTGTGCGTGACA<br>TGAGGCAGACCGTTGCTGTTGGTGTCATCAAGAGCGTGGAGAAGG
18S	GTTCTTAGTTGGTGGAGCGATTTGTCTGGTTAATTCCGTTAACGAACGAGACCTCAGCC<br>TGCTAACTAGCTATGCGGAGCCATCCCTCCGCAGCTAGCTTCTTAGAGGGACTATCGCC<br>GTTTAG
25S	CCACTGTCCCTGTCTACTATCCAGCGAAACCACAGCCAAGGGAACGGGCTTGGCGGA<br>ATCAGCGGGGAAAGAAGACCCTGTTGAGCTTGACTCTAGTCCGACTTTGTGAAATGAC<br>TTGAGAGGTGTAGGATAAGTGGGAG
RUBP	TGCCAGCTCTGACCGAAATCTTTGGGGATGATTCTGTATTACAATTTGGTGGAGGAACT<br>TTAGGACATCCTTGGGGAAATGCACCTGGTGCAGCAGCTAATCGAGTGGCTTTAGAAG<br>CCTGTGTACAAGCTCGTAACGAAGGGCGCGATCTTGCTCGTGAAGGTAATGAAATTAT<br>CCGAGCAGCTTGCAAATGGAGTCCTGAACTAGCCGC
GAPDH	CCACTAACTGCCTTGCTCCTCTTGCTAAGGTTATCAATGACAAGTTTGGCATCGTTGAG<br>GGTTTGATGACCACTGTTCATGCCATGACCGCAACCCAGAAGACTGTTGATGGTCCCT<br>CAAGCAAGGACTGGAGAGGTGGAAGGGCTGCTAGCTTCAACATCATTCCCAGCAGCA<br>CTGGAGCTGCCAAGGCTGTTGGCAAGGTGCTTCCAGTCCTTAACGGAAAGTTGACAG<br>GAATGGCCTTCCGTGTTCCAACCGTTGATGT

Table S2. Putative P450 genes/alleles in *P. fugax* transcriptome assembled.

ID	Nr-ID	Nr-Score	Nr-E value	Nr-annotation
Isoform0008742	KAE8806524.1	438	0	protein CYPRO4-like [Hordeum vulgare]
Isoform0010815	KAE8806524.1	1102	0	protein CYPRO4-like [Hordeum vulgare]
Isoform0011097	KAE8806524.1	453	3.00E-148	protein CYPRO4-like [Hordeum vulgare]
Isoform0011977	KAE8806524.1	601	0	protein CYPRO4-like [Hordeum vulgare]
Isoform0013125	KAE8806524.1	799	0	protein CYPRO4-like [Hordeum vulgare]
Isoform0011103	XP_020149134.1	711	0	probable cytochrome P450 313a4 [Aegilops tauschii subsp. tauschii] [Aegilops tauschii]
Isoform0005613	XP_020191364.1	652	0	peptidyl-prolyl cis-trans isomerase CYP95 isoform X4 [Aegilops tauschii subsp. tauschii] [Aegilops tauschii]
Isoform0005631	XP_020191364.1	651	0	peptidyl-prolyl cis-trans isomerase CYP95 isoform X4 [Aegilops tauschii subsp. tauschii] [Aegilops tauschii]
Isoform0005740	XP_020191364.1	280	2.00E-131	peptidyl-prolyl cis-trans isomerase CYP95 isoform X4 [Aegilops tauschii subsp. tauschii] [Aegilops tauschii]
Isoform0006136	XP_020191364.1	653	0	peptidyl-prolyl cis-trans isomerase CYP95 isoform X4 [Aegilops tauschii subsp. tauschii] [Aegilops tauschii]
Isoform0006387	XP_020191364.1	571	0	peptidyl-prolyl cis-trans isomerase CYP95 isoform X4 [Aegilops tauschii subsp. tauschii] [Aegilops tauschii]
Isoform0007370	XP_020191364.1	610	0	peptidyl-prolyl cis-trans isomerase CYP95 isoform X4 [Aegilops tauschii subsp. tauschii] [Aegilops tauschii]
Isoform0005938	XP_010234011.1	1012	0	peptidyl-prolyl cis-trans isomerase CYP95 isoform X3 [Brachypodium distachyon]
Isoform0006890	XP_010234010.1	1018	0	peptidyl-prolyl cis-trans isomerase CYP95 isoform X2 [Brachypodium distachyon]
Isoform0004991	XP_020191362.1	564	0	peptidyl-prolyl cis-trans isomerase CYP95 isoform X2 [Aegilops tauschii subsp. tauschii] [Aegilops tauschii]
Isoform0010638	XP_025800948.1	320	7.00E-158	peptidyl-prolyl cis-trans isomerase CYP63-like isoform X1 [Panicum hallii]
Isoform0016167	KAE8799446.1	849	0	peptidyl-prolyl cis-trans isomerase CYP57 [Hordeum vulgare]
Isoform0018636	XP_003562877.1	776	0	peptidyl-prolyl cis-trans isomerase CYP37, chloroplastic isoform X2 [Brachypodium distachyon]
Isoform0018841	PWZ41636.1	402	0	Peptidyl-prolyl cis-trans isomerase CYP37, chloroplastic [Zea mays]
Isoform0003294	XP_003573666.2	566	0	peptidyl-prolyl cis-trans isomerase CYP28, chloroplastic isoform X1 [Brachypodium distachyon]
Isoform0004028	XP_003573666.2	573	0	peptidyl-prolyl cis-trans isomerase CYP28, chloroplastic isoform X1 [Brachypodium distachyon]
Isoform0020133	XP_003573666.2	570	0	peptidyl-prolyl cis-trans isomerase CYP28, chloroplastic isoform X1 [Brachypodium distachyon]
Isoform0023932	XP_003563897.1	393	3.00E-137	peptidyl-prolyl cis-trans isomerase CYP22 [Brachypodium distachyon]
Isoform0022093	XP_015647974.1	423	3.00E-147	peptidyl-prolyl cis-trans isomerase CYP21-4 [Oryza sativa Japonica Group]
Isoform0023611	XP_010227456.1	387	3.00E-134	peptidyl-prolyl cis-trans isomerase CYP20-1 isoform X2 [Brachypodium distachyon]
Isoform0024031	XP_010227456.1	384	2.00E-133	peptidyl-prolyl cis-trans isomerase CYP20-1 isoform X2 [Brachypodium distachyon]

Isoform0024098	XP_010227456.1	386	2.00E-134	peptidyl-prolyl cis-trans isomerase CYP20-1 isoform X2 [Brachypodium distachyon]
Isoform0010977	BAD27508.1	557	0	P450 [Lolium rigidum]
Isoform0014118	BAD27508.1	937	0	P450 [Lolium rigidum]
Isoform0014911	BAD27508.1	949	0	P450 [Lolium rigidum]
Isoform0017326	BAD27508.1	909	0	P450 [Lolium rigidum]
Isoform0023558	BAD27508.1	479	7.00E-166	P450 [Lolium rigidum]
Isoform0012489	XP_020186523.1	1155	0	NADPH--cytochrome P450 reductase-like [Aegilops tauschii subsp. tauschii] [Aegilops tauschii]
Isoform0014834	XP_020186523.1	1029	0	NADPH--cytochrome P450 reductase-like [Aegilops tauschii subsp. tauschii] [Aegilops tauschii]
Isoform0007596	EMS66177.1	1148	0	NADPH--cytochrome P450 reductase [Triticum urartu]
Isoform0008041	XP_015651232.1	1241	0	NADPH--cytochrome P450 reductase [Oryza sativa Japonica Group]
Isoform0008211	XP_015651232.1	1236	0	NADPH--cytochrome P450 reductase [Oryza sativa Japonica Group]
Isoform0009009	XP_015651232.1	1231	0	NADPH--cytochrome P450 reductase [Oryza sativa Japonica Group]
Isoform0013133	XP_015650780.1	1117	0	NADPH--cytochrome P450 reductase [Oryza sativa Japonica Group]
Isoform0007794	XP_010234513.1	1280	0	NADPH--cytochrome P450 reductase [Brachypodium distachyon]
Isoform0015082	ABG88966.1	587	0	cytochrome P450 CYP51H11 [Avena strigosa]
Isoform0015491	KAE8819785.1	728	0	Cytochrome P450 99A2 [Hordeum vulgare]
Isoform0017350	KAE8819785.1	750	0	Cytochrome P450 99A2 [Hordeum vulgare]
Isoform0009151	EMS51606.1	1128	0	Cytochrome P450 97B2, chloroplastic [Triticum urartu]
Isoform0008960	KAE8774286.1	835	0	Cytochrome P450 97B2, chloroplastic [Hordeum vulgare]
Isoform0009725	KAE8774286.1	1092	0	Cytochrome P450 97B2, chloroplastic [Hordeum vulgare]
Isoform0010975	KAE8774286.1	1080	0	Cytochrome P450 97B2, chloroplastic [Hordeum vulgare]
Isoform0012522	KAE8774286.1	1080	0	Cytochrome P450 97B2, chloroplastic [Hordeum vulgare]
Isoform0013160	KAE8774286.1	1093	0	Cytochrome P450 97B2, chloroplastic [Hordeum vulgare]
Isoform0014768	KAE8774286.1	1094	0	Cytochrome P450 97B2, chloroplastic [Hordeum vulgare]
Isoform0015603	KAE8786242.1	915	0	cytochrome P450 94C1-like [Hordeum vulgare]
Isoform0016999	KAE8786242.1	914	0	cytochrome P450 94C1-like [Hordeum vulgare]
Isoform0016252	XP_020201297.1	919	0	cytochrome P450 94C1-like [Aegilops tauschii subsp. tauschii] [Aegilops tauschii]
Isoform0014994	XP_020192548.1	867	0	cytochrome P450 94B3-like [Aegilops tauschii subsp. tauschii] [Aegilops tauschii]

Isoform0015013	XP_020192548.1	859	0	cytochrome P450 94B3-like [Aegilops tauschii subsp. tauschii] [Aegilops tauschii]
Isoform0016000	XP_020192546.1	857	0	cytochrome P450 94B3-like [Aegilops tauschii subsp. tauschii] [Aegilops tauschii]
Isoform0017486	XP_020192546.1	853	0	cytochrome P450 94B3-like [Aegilops tauschii subsp. tauschii] [Aegilops tauschii]
Isoform0016309	KAE8806732.1	859	0	cytochrome P450 94B3 [Hordeum vulgare]
Isoform0017202	KAE8806732.1	767	0	cytochrome P450 94B3 [Hordeum vulgare]
Isoform0017403	XP_003577084.3	888	0	cytochrome P450 94B3 [Brachypodium distachyon]
Isoform0014309	XP_020148182.1	570	0	cytochrome P450 94A2-like [Aegilops tauschii subsp. tauschii] [Aegilops tauschii]
Isoform0016504	XP_020148182.1	911	0	cytochrome P450 94A2-like [Aegilops tauschii subsp. tauschii] [Aegilops tauschii]
Isoform0016535	XP_020191526.1	919	0	cytochrome P450 93A3-like [Aegilops tauschii subsp. tauschii] [Aegilops tauschii]
Isoform0015154	XP_020148060.1	884	0	cytochrome P450 93A2-like [Aegilops tauschii subsp. tauschii] [Aegilops tauschii]
Isoform0015211	XP_020148060.1	882	0	cytochrome P450 93A2-like [Aegilops tauschii subsp. tauschii] [Aegilops tauschii]
Isoform0015698	XP_020160430.1	929	0	cytochrome P450 90A1 [Aegilops tauschii subsp. tauschii] [Aegilops tauschii]
Isoform0011236	XP_020174452.1	328	2.00E-101	cytochrome P450 89A2-like [Aegilops tauschii subsp. tauschii] [Aegilops tauschii]
Isoform0014630	XP_020169689.1	839	0	cytochrome P450 89A2-like [Aegilops tauschii subsp. tauschii] [Aegilops tauschii]
Isoform0014806	XP_020169689.1	847	0	cytochrome P450 89A2-like [Aegilops tauschii subsp. tauschii] [Aegilops tauschii]
Isoform0016001	XP_020169689.1	839	0	cytochrome P450 89A2-like [Aegilops tauschii subsp. tauschii] [Aegilops tauschii]
Isoform0016011	XP_020185943.1	913	0	cytochrome P450 89A2-like [Aegilops tauschii subsp. tauschii] [Aegilops tauschii]
Isoform0016233	XP_020192380.1	615	0	cytochrome P450 89A2-like [Aegilops tauschii subsp. tauschii] [Aegilops tauschii]
Isoform0016523	XP_020192380.1	618	0	cytochrome P450 89A2-like [Aegilops tauschii subsp. tauschii] [Aegilops tauschii]
Isoform0016839	XP_020169689.1	846	0	cytochrome P450 89A2-like [Aegilops tauschii subsp. tauschii] [Aegilops tauschii]
Isoform0016914	XP_020185943.1	877	0	cytochrome P450 89A2-like [Aegilops tauschii subsp. tauschii] [Aegilops tauschii]
Isoform0016965	XP_020185943.1	868	0	cytochrome P450 89A2-like [Aegilops tauschii subsp. tauschii] [Aegilops tauschii]
Isoform0016977	XP_020192380.1	760	0	cytochrome P450 89A2-like [Aegilops tauschii subsp. tauschii] [Aegilops tauschii]
Isoform0016988	XP_020192380.1	615	0	cytochrome P450 89A2-like [Aegilops tauschii subsp. tauschii] [Aegilops tauschii]
Isoform0017103	XP_020169689.1	847	0	cytochrome P450 89A2-like [Aegilops tauschii subsp. tauschii] [Aegilops tauschii]
Isoform0017816	XP_020169689.1	835	0	cytochrome P450 89A2-like [Aegilops tauschii subsp. tauschii] [Aegilops tauschii]
Isoform0018303	XP_020192380.1	615	0	cytochrome P450 89A2-like [Aegilops tauschii subsp. tauschii] [Aegilops tauschii]
Isoform0018634	XP_020185943.1	834	0	cytochrome P450 89A2-like [Aegilops tauschii subsp. tauschii] [Aegilops tauschii]

Isoform0022201	XP_020192380.1	454	2.00E-155	cytochrome P450 89A2-like [Aegilops tauschii subsp. tauschii] [Aegilops tauschii]
Isoform0001085	EMS58082.1	489	5.00E-157	Cytochrome P450 89A2 [Triticum urartu]
Isoform0016809	EMS58082.1	806	0	Cytochrome P450 89A2 [Triticum urartu]
Isoform0017802	EMS58082.1	795	0	Cytochrome P450 89A2 [Triticum urartu]
Isoform0020157	KAE8787333.1	559	0	Cytochrome P450 89A2 [Hordeum vulgare]
Isoform0015705	XP_003579360.1	187	6.00E-93	cytochrome P450 89A2 [Brachypodium distachyon]
Isoform0013450	XP_020173163.1	1030	0	cytochrome P450 86B1-like [Aegilops tauschii subsp. tauschii] [Aegilops tauschii]
Isoform0015066	XP_020169928.1	936	0	cytochrome P450 86B1-like [Aegilops tauschii subsp. tauschii] [Aegilops tauschii]
Isoform0015286	XP_020169928.1	932	0	cytochrome P450 86B1-like [Aegilops tauschii subsp. tauschii] [Aegilops tauschii]
Isoform0016534	XP_020169928.1	939	0	cytochrome P450 86B1-like [Aegilops tauschii subsp. tauschii] [Aegilops tauschii]
Isoform0016777	EMS50899.1	766	0	Cytochrome P450 86B1 [Triticum urartu]
Isoform0018582	EMS50899.1	766	0	Cytochrome P450 86B1 [Triticum urartu]
Isoform0021166	EMS50900.1	621	0	Cytochrome P450 86B1 [Triticum urartu]
Isoform0021992	EMS50899.1	212	8.00E-130	Cytochrome P450 86B1 [Triticum urartu]
Isoform0012772	XP_020189682.1	900	0	cytochrome P450 84A1-like [Aegilops tauschii subsp. tauschii] [Aegilops tauschii]
Isoform0014296	XP_020189682.1	901	0	cytochrome P450 84A1-like [Aegilops tauschii subsp. tauschii] [Aegilops tauschii]
Isoform0012689	XP_020167440.1	933	0	cytochrome P450 78A9-like [Aegilops tauschii subsp. tauschii] [Aegilops tauschii]
Isoform0018287	KAE8786115.1	807	0	Cytochrome P450 76C4 [Hordeum vulgare]
Isoform0018408	KAE8786115.1	791	0	Cytochrome P450 76C4 [Hordeum vulgare]
Isoform0018802	KAE8786115.1	484	0	Cytochrome P450 76C4 [Hordeum vulgare]
Isoform0018872	KAE8786115.1	790	0	Cytochrome P450 76C4 [Hordeum vulgare]
Isoform0013727	KAE8782841.1	947	0	Cytochrome P450 76C1 [Hordeum vulgare]
Isoform0013892	KAE8782841.1	946	0	Cytochrome P450 76C1 [Hordeum vulgare]
Isoform0013956	KAE8782841.1	941	0	Cytochrome P450 76C1 [Hordeum vulgare]
Isoform0014497	KAE8782841.1	946	0	Cytochrome P450 76C1 [Hordeum vulgare]
Isoform0015349	KAE8782841.1	947	0	Cytochrome P450 76C1 [Hordeum vulgare]
Isoform0015575	KAE8782841.1	942	0	Cytochrome P450 76C1 [Hordeum vulgare]
Isoform0015673	KAE8782841.1	946	0	Cytochrome P450 76C1 [Hordeum vulgare]

Isoform0016629	KAE8782841.1	946	0	Cytochrome P450 76C1 [Hordeum vulgare]
Isoform0017904	KAE8782841.1	885	0	Cytochrome P450 76C1 [Hordeum vulgare]
Isoform0020327	KAE8782841.1	825	0	Cytochrome P450 76C1 [Hordeum vulgare]
Isoform0012648	XP_010227548.1	500	0	cytochrome P450 734A4 [Brachypodium distachyon]
Isoform0015706	XP_003568484.1	437	0	cytochrome P450 734A1 [Brachypodium distachyon]
Isoform0013922	XP_003569397.1	975	0	cytochrome P450 72A15 [Brachypodium distachyon]
Isoform0015141	XP_003569397.1	981	0	cytochrome P450 72A15 [Brachypodium distachyon]
Isoform0015640	XP_003569397.1	975	0	cytochrome P450 72A15 [Brachypodium distachyon]
Isoform0016150	XP_003566917.1	764	0	cytochrome P450 72A15 [Brachypodium distachyon]
Isoform0016181	XP_003566917.1	764	0	cytochrome P450 72A15 [Brachypodium distachyon]
Isoform0016325	XP_003569397.1	933	0	cytochrome P450 72A15 [Brachypodium distachyon]
Isoform0016830	XP_003569397.1	940	0	cytochrome P450 72A15 [Brachypodium distachyon]
Isoform0015806	EMS54343.1	834	0	Cytochrome P450 71D8 [Triticum urartu]
Isoform0013902	XP_020148443.1	364	0	cytochrome P450 71A1-like [Aegilops tauschii subsp. tauschii] [Aegilops tauschii]
Isoform0014269	XP_020148443.1	536	0	cytochrome P450 71A1-like [Aegilops tauschii subsp. tauschii] [Aegilops tauschii]
Isoform0014779	XP_020199217.1	749	0	cytochrome P450 71A1-like [Aegilops tauschii subsp. tauschii] [Aegilops tauschii]
Isoform0014972	XP_020164316.1	801	0	cytochrome P450 71A1-like [Aegilops tauschii subsp. tauschii] [Aegilops tauschii]
Isoform0015736	XP_020199217.1	753	0	cytochrome P450 71A1-like [Aegilops tauschii subsp. tauschii] [Aegilops tauschii]
Isoform0015962	XP_020192280.1	725	0	cytochrome P450 71A1-like [Aegilops tauschii subsp. tauschii] [Aegilops tauschii]
Isoform0016055	XP_020164316.1	801	0	cytochrome P450 71A1-like [Aegilops tauschii subsp. tauschii] [Aegilops tauschii]
Isoform0016124	XP_020192280.1	723	0	cytochrome P450 71A1-like [Aegilops tauschii subsp. tauschii] [Aegilops tauschii]
Isoform0016212	XP_020192280.1	758	0	cytochrome P450 71A1-like [Aegilops tauschii subsp. tauschii] [Aegilops tauschii]
Isoform0016735	XP_020192280.1	484	0	cytochrome P450 71A1-like [Aegilops tauschii subsp. tauschii] [Aegilops tauschii]
Isoform0016799	XP_020199217.1	749	0	cytochrome P450 71A1-like [Aegilops tauschii subsp. tauschii] [Aegilops tauschii]
Isoform0017108	XP_020199217.1	700	0	cytochrome P450 71A1-like [Aegilops tauschii subsp. tauschii] [Aegilops tauschii]
Isoform0017200	XP_020192280.1	561	0	cytochrome P450 71A1-like [Aegilops tauschii subsp. tauschii] [Aegilops tauschii]
Isoform0017233	XP_020182992.1	318	2.00E-135	cytochrome P450 71A1-like [Aegilops tauschii subsp. tauschii] [Aegilops tauschii]
Isoform0017416	XP_020192280.1	613	0	cytochrome P450 71A1-like [Aegilops tauschii subsp. tauschii] [Aegilops tauschii]

Isoform0017466	XP_020199217.1	749	0	cytochrome P450 71A1-like [Aegilops tauschii subsp. tauschii] [Aegilops tauschii]
Isoform0017567	XP_020192280.1	317	2.00E-99	cytochrome P450 71A1-like [Aegilops tauschii subsp. tauschii] [Aegilops tauschii]
Isoform0018871	XP_020199217.1	746	0	cytochrome P450 71A1-like [Aegilops tauschii subsp. tauschii] [Aegilops tauschii]
Isoform0016508	KAE8817588.1	308	0	Cytochrome P450 71A1 [Hordeum vulgare]
Isoform0018098	KAE8817588.1	446	0	Cytochrome P450 71A1 [Hordeum vulgare]
Isoform0007863	XP_003563018.4	721	0	cytochrome P450 716B1 [Brachypodium distachyon]
Isoform0014130	XP_003563018.4	423	0	cytochrome P450 716B1 [Brachypodium distachyon]
Isoform0015165	XP_003563018.4	819	0	cytochrome P450 716B1 [Brachypodium distachyon]
Isoform0015365	XP_003563018.4	812	0	cytochrome P450 716B1 [Brachypodium distachyon]
Isoform0014751	XP_020199577.1	624	0	cytochrome P450 714C2-like isoform X1 [Aegilops tauschii subsp. tauschii] [Aegilops tauschii]
Isoform0012562	XP_020183387.1	374	2.00E-119	cytochrome P450 711A1-like [Aegilops tauschii subsp. tauschii] [Aegilops tauschii]
Isoform0014981	XP_015648135.1	885	0	cytochrome P450 710A1 [Oryza sativa Japonica Group]
Isoform0016146	XP_015648135.1	889	0	cytochrome P450 710A1 [Oryza sativa Japonica Group]
Isoform0015014	XP_003578542.1	639	0	cytochrome P450 709B2 isoform X1 [Brachypodium distachyon]
Isoform0015351	XP_003578542.1	426	1.00E-145	cytochrome P450 709B2 isoform X1 [Brachypodium distachyon]
Isoform0016740	XP_003557875.1	880	0	cytochrome P450 709B2 [Brachypodium distachyon]
Isoform0016051	XP_020174721.1	961	0	cytochrome P450 709B1-like [Aegilops tauschii subsp. tauschii] [Aegilops tauschii]
Isoform0018862	XP_020184145.1	897	0	cytochrome P450 709B1-like [Aegilops tauschii subsp. tauschii] [Aegilops tauschii]
Isoform0016911	XP_020189681.1	883	0	cytochrome P450 704C1-like [Aegilops tauschii subsp. tauschii] [Aegilops tauschii]
Isoform0015291	AAG17469.1	328	0	cytochrome P450 [Triticum aestivum]
Isoform0014862	CCP37680.1	845	0	cytochrome P450 [Lolium sp. CD-2012]
Isoform0015679	CCP37680.1	853	0	cytochrome P450 [Lolium sp. CD-2012]
Isoform0016144	CCP37680.1	848	0	cytochrome P450 [Lolium sp. CD-2012]
Isoform0016463	CCP37680.1	853	0	cytochrome P450 [Lolium sp. CD-2012]
Isoform0017144	CCP37680.1	853	0	cytochrome P450 [Lolium sp. CD-2012]
Isoform0021165	BAF63628.1	572	0	cytochrome P450 [Lolium rigidum]
Isoform0016936	KAE8773448.1	659	0	cytochrome P450 [Hordeum vulgare]
Isoform0015662	AER39772.1	997	0	CYP92A44-2 [Festuca rubra subsp. commutata] [Festuca rubra]

Isoform0015832	AER39772.1	985	0	CYP92A44-2 [Festuca rubra subsp. commutata] [Festuca rubra]
Isoform0016221	AER39772.1	801	0	CYP92A44-2 [Festuca rubra subsp. commutata] [Festuca rubra]
Isoform0013399	CDO33950.1	907	0	CYP450 [Hordeum vulgare]

Table S3. Primers used for P450 gene expression assay.

Gene ID	Isoform	Annotation	Primer	Sequence (5' to 3')
P450-1	Isoform0008742	CYPRO4	F	GAGTGGCGGTTCGAGAAGGATG
	Isoform0010815		R	AAGAAGGTTGACCCGGAGGGATC
	Isoform0011097			
	Isoform0011977			
	Isoform0013125			
P450-2	Isoform0011103	CYP313a4	F	GTCAACTGTACTGTGGACAAGGACTC
			R	CAGCAGCATCAGACCAATCAAAGAAG
P450-3	Isoform0005613	CYP95	F	AAACTTTGTGCTATGCCATGCTGAAC
	Isoform0005631		R	TGAAGAACTGGGAGCCATTGGTATTAC
	Isoform0005740			
	Isoform0006136			
	Isoform0006387			
	Isoform0007370			
	Isoform0005938			
	Isoform0006890			
	Isoform0004991			
P450-4	Isoform0010638	CYP63	F	AACTCTGGAGAAAACACCAACGGATC
			R	AAGCACCTTCCCAAAGACCACATG
P450-5	Isoform0018841	CYP57	F	TTGAACAACTGTCCAAGGAGGATGAG
			R	CGTTGTCGTCGTCAGGAGAATCG
P450-6	Isoform0018841	CYP37	F	TTCCGATGTCTGTATATGGCTCTGTTG
	Isoform0018636		R	GGTAGAAGAAGAACTGGGTTGGTGATG
P450-7	Isoform0003294	CYP28	F	TTGGTCTCTATGGTCGGCTACTCC
	Isoform0004028		R	GGACAAGCGTGCCTCGATATGC
	Isoform0020133			
P450-8	Isoform0023932	CYP22	F	GCGGAAGATTGAGAATGTAGCAACTG
			R	TTACATCTCACCACACTCGCTGATAAC
P450-9	Isoform0022093	CYP21-4	F	CGACCTACAGGCATTGGCATCAC
			R	TGAGCCTTTCGCAGTGTCCATTAC
P450-10	Isoform0023611	CYP20-1	F	ACTTCAAGCTCAAGCACACTGGAC
	Isoform0024031		R	AGAGATCACCTTGCCGAACACAAC
	Isoform0024098			
P450-11	Isoform0010977		F	CATGTCGCTCCTGCTGAACCAC
	Isoform0014118		R	AGCGTCTCGTTGATGATGCACTG
	Isoform0014911			
	Isoform0017326			
	Isoform0023558			
P450-12	Isoform0012489		F	TCCACCATTCCCATCTCCTTCTACC
	Isoform0014834		R	AAACCTCAGTCGTTCAGCTTCAGTC
P450-13	Isoform0007596		F	TAGGCAGTACGAGCATTTCAACAAGG
	Isoform0008041		R	CATCTCCAAGACCAACAGGAACTAGG
	Isoform0008211			

	Isoform0013133			
	Isoform0007794			
P450-14	Isoform0015082	CYP51H11	F	GTCGTGGCACAACCGTAGAAGAG
			R	TTTGCTCCTCTAAAGCGGCATCTAAG
P450-15	Isoform0015491	CYP99A2	F	AAGCCTCCTCCACCTCCTCA
	Isoform0017350		R	AGACGCAGGTACATCACGGG
P450-16	Isoform0009151	CYP97B2	F	ATTTGGCTCTTGACATAATTGGCTTGG
	Isoform0008960		R	AAAGGTGGATCGATGCTCAGCTTC
	Isoform0009725			
	Isoform0010975			
	Isoform0012522			
	Isoform0013160			
	Isoform0014768			
P450-17	Isoform0015603	CYP94C1	F	GCCAACTCGGAGAAACACCC
	Isoform0016999		R	GCGACGAGACGGAAGCAGTA
	Isoform0016252			
P450-18-1	Isoform0014994	CYP94B3-1	F	GTCCCTGCTCCACGACTTCCTC
	Isoform0015013		R	GAACTCGTAGCTGGCGGTCTTG
	Isoform0016000			
	Isoform0017486			
P450-18-2	Isoform0016309	CYP94B3-2	F	GACGTGGTCACCAACTTCATCCTC
	Isoform0017202		R	GTGGACACCAGCCAGAAGAACC
P450-18-3	Isoform0017403	CYP94B3-3	F	CGCTCCGGGACATGCATTAC
			R	TGTACGTCACGATCCAGCCC
P450-19	Isoform0014309	CYP94A2	F	ACCCAGCGACGGCCTCCCTCCTCGC
	Isoform0016504		R	GCGAGGAGGGAGGCCGTCGCTGGGT
	Isoform0016535			
	Isoform0015154			
	Isoform0015211			
P450-20	Isoform0015698	CYP90A1	F	CTCATCGACGGCTTCTTCTCCATC
			R	TCCTCCATCCTCTTCCTTATCACTTCC
P450-21	Isoform0011236	CYP89A2	F	CCACGAACAGACGCAGCAGGAAGCG
	Isoform0014630		R	CGCTTCCTGCTGCGTCTGTTCGTGG
	Isoform0014806			
	Isoform0016001			
	Isoform0016011			
	Isoform0016233			
	Isoform0016523			
	Isoform0016839			
	Isoform0016914			
	Isoform0016965			
	Isoform0016977			
	Isoform0016988			
	Isoform0017103			

	Isoform0017816			
	Isoform0018303			
	Isoform0018634			
	Isoform0022201			
	Isoform0001085			
	Isoform0016809			
	Isoform0017802			
	Isoform0020157			
	Isoform0015705			
P450-22	Isoform0013450	CYP86B1	F	GTCTTGGGCAACGGTATCTTCA
	Isoform0015066		R	TCCACCTTCTGGCGGCTGTA
	Isoform0015286			
	Isoform0016534			
	Isoform0016777			
	Isoform0018582			
	Isoform0021166			
	Isoform0021992			
P450-23	Isoform0012772	CYP84A1	F	ACGATGGAGGGAAGCAGGAC
	Isoform0014296		R	GTCCTGCTCAAGTAGCGGTA
P450-24	Isoform0012689	CYP78A9	F	CTCGTCCCCAGAGTCAATCATTTCG
			R	GTCGGCGTCTGAGAGTTTGTCAC
P450-25	Isoform0018287	CYP76C4	F	CCCAACGTCTCCGACTTCTTT
	Isoform0018408		R	AATGCGGCGATCAATTATGC
	Isoform0018802			
	Isoform0018872			
P450-26	Isoform0013727	CYP76C1	F	GTCAAGGCCCTGCTCATGGATATG
	Isoform0013892		R	CCACCGCATCTAGCTCCTCCTG
	Isoform0013892			
	Isoform0013956			
	Isoform0014497			
	Isoform0015349			
	Isoform0015575			
	Isoform0015673			
	Isoform0016629			
	Isoform0017904			
	Isoform0020327			
P450-27	Isoform0012648	CYP734A4	F	ACAACGAAACGAAGACCAACAACAAG
			R	TGCACTCCTCCACCATGTCCTC
P450-28	Isoform0015706	CYP734A1	F	GCTATCACCTTGGTGCGTACTTCC
			R	AAGTGCCAGCTTTGCCTCAACC
P450-29-1	Isoform0013922	CYP72A15-1	F	GGCCCGAGTTCCAGAATCTTACG
	Isoform0015141		R	AGCTGGAATATTTTCATCCCCTCCTG
P450-29-2	Isoform0016150	CYP72A15-2	F	TTAAATCGCCTGAGAACCGTGAGC
	Isoform0016181		R	CCTAACGCCGTCGAGATCCATTG

P450-30	Isoform0015806	CYP71D8	F	GATCACTGGCGTGACATGCGTAAG
			R	CTGATGCTCTCCATGCGCTTCAC
P450-31-1	Isoform0014972	CYP71A1-1	F	GCGGACCTATACAAGAACCACGAC
	Isoform0014269		R	GAAGGAGATGTTGTTGGAGCCGTAG
	Isoform0015962			
	Isoform0016124			
P450-31-2	Isoform0014972	CYP71A1-2	F	CGGTGTGCTGGAGAAGGTGATC
	Isoform0016055		R	TTCTTTGTCGGTCTCGTTCACTTCC
P450-31-3	Isoform0014779	CYP71A1-3	F	AGACCCAGGACCACGTCTTCTG
	Isoform0015736		R	CCGCTCTCCTTAGGGGCTGAAG
	Isoform0016799			
	Isoform0018871			
	Isoform0018098			
	Isoform0017466			
	Isoform0017233			
P450-31-4	Isoform0016212	CYP71A1-4	F	TACAGGACGAGGGTAACACGAGTC
	Isoform0016735		R	CCTTCAGCACCGCCTTGAGATAC
	Isoform0017200			
	Isoform0017416			
	Isoform0017567			
P450-31-5	Isoform0017108	CYP71A1-5	F	CCTACAAGACGATAGAATGGACGATGG
	Isoform0016508		R	CAGCAACCTGTCTCACCTCTGATTG
P450-32	Isoform0007863	CYP716B1	F	GGCCAACAAGTTCATCTTCTTCAACAG
	Isoform0014130		R	TGCTCTTCTCACCGACTATCCTCTG
	Isoform0015165			
	Isoform0015365			
P450-33	Isoform0014751	CYP714C2	F	GAGTATGCTTATTGGTGTTGCTGGAAG
			R	AATGAGGGTACGGATGCTGCTTTC
P450-34	Isoform0012562	CYP711A1	F	ATCCTCGGGCTATTCCTCCC
			R	CATCTTGTAGTCCGCCGTGC
P450-35	Isoform0014981	CYP710A1	F	CATCGTCTTCATCCGCGACTCC
	Isoform0016146		R	GTCCTTGTGGTCATCGCCGAAC
P450-36	Isoform0015014	CYP709B2	F	CTCCGACTTCTTTCCTTTCCTCC
	Isoform0015351		R	CTCCGACTTCTTTCCTTTCCTCC
P450-37	Isoform0016051	CYP709B1	F	GCAGGCTCACATCCACAAATG
	Isoform0018862		R	GCCCCGAACCAGTACAGGAA
P450-38	Isoform0016911	CYP704C1	F	GTACCTGCGGGACATCATACTGA
			R	GTCCTGGATGTGCTGGTTGC
P450-39	Isoform0015291		F	ATCGGCTCCCATGACTTCG
	Isoform0014862		R	TTTGGGTACAGCCCGTTGC
	Isoform0015679			
	Isoform0016144			
	Isoform0016463			
	Isoform0017144			

	Isoform0021165			
	Isoform0016936			
P450-40	Isoform0015662	CYP92A44-2	F	GCTCAACATCGGCGACTCCA
	Isoform0015832		R	CGAACATCTTGCTCAGCTTCTTCAT
	Isoform0016221			
P450-41	Isoform0013399		F	GCAAGACGCGTATGGTGGTG
			R	CGGTGGAACAAGAGCCCGTA

Table S4. Ranking of seven candidate reference genes according to their expression stability value (SV) in different data subsets as computed using NormFinder.

Rank	All-samples set		Fenoxaprop-*P*-ethyl subset				Mesosulfuron-methyl subset				Growth-stage subset		Organ subset	
			No group		Phenotype groups		No group		Phenotype groups					
	Gene	SV	Gene	SV	Gene	SV	Gene	SV	Gene	SV	Gene	SV	Gene	SV
1	*EIF4A*	1.870	*ACT*	0.426	*EF1α*	0.235	*GAPDH*	0.533	*GAPDH*	0.566	*25S*	0.507	*RUBP*	1.049
2	*EF1α*	1.023	*EF1α*	0.469	*RUBP*	0.315	*RUBP*	0.748	*RUBP*	0.637	*GAPDH*	0.544	*EF1α*	1.349
3	*18S*	1.451	*18S*	0.691	*ACT*	0.316	*ACT*	0.970	*EF1α*	0.972	*EF1α*	0.568	*EIF4A*	2.016
4	*25S*	2.723	*RUBP*	0.699	*18S*	0.323	*EF1α*	1.049	*ACT*	0.999	*EIF4A*	0.704	*18S*	2.055
5	*RUBP*	1.821	*25S*	1.220	*25S*	0.517	*EIF4A*	1.489	*EIF4A*	1.213	*ACT*	0.805	*GAPDH*	2.545
6	*GAPDH*	1.512	*EIF4A*	1.269	*EIF4A*	0.556	*18S*	1.712	*18S*	1.293	*18S*	0.810	*25S*	4.846
7	*ACT*	2.540	*GAPDH*	1.778	*GAPDH*	0.682	*25S*	2.781	*25S*	1.828	*RUBP*	1.028	*ACT*	5.678
Combination	-	-	-	-	*EF1α*, *18S*	0.205	-	-	*ACT, 18S*	0.493	-	-	-	-

Table S5. Ranking of seven candidate reference genes according to their stability of expression using the comparative ΔCq method.

Rank	All-samples set		Fenoxaprop-*P*-ethyl subset		Mesosulfuron-methyl subset		Growth stage subset		Organ subset	
	Gene	AS	Gene	AS	Gene	AS	Gene	AS	Gene	AS
1	*EF1α*	3.24	*ACT*	1.63	*GAPDH*	2.11	*25S*	1.78	*RUBP*	4.93
2	*18S*	3.43	*18S*	1.67	*EF1α*	2.28	*EF1α*	2.21	*EF1α*	5.03
3	*GAPDH*	3.49	*EF1α*	1.68	*ACT*	2.32	*EIF4A*	2.63	*18S*	5.2
4	*RUBP*	3.79	*RUBP*	1.75	*RUBP*	2.36	*ACT*	3.5	*EIF4A*	5.53
5	*EIF4A*	3.79	*25S*	2.07	*EIF4A*	2.64	*GAPDH*	3.6	*GAPDH*	5.79
6	*ACT*	4.35	*EIF4A*	2.28	*18S*	3.15	*RUBP*	5.66	*25S*	7.59
7	*25S*	4.48	*GAPDH*	2.82	*25S*	4.17	*18S*	5.73	*ACT*	8.71

AS, Average of STDEV.

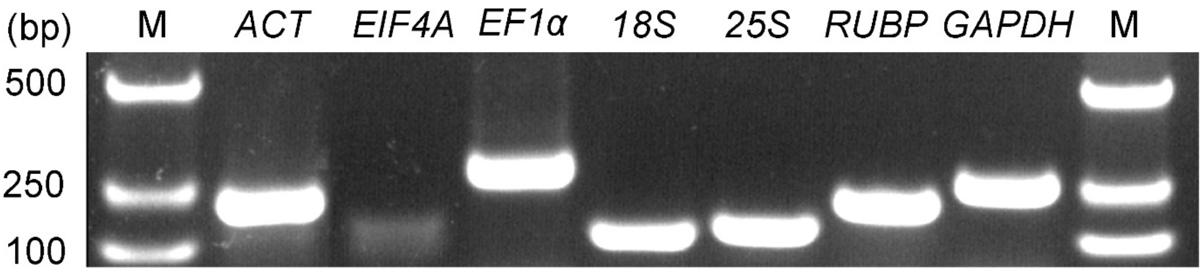

Fig. S1. Agarose gel electrophoresis showing amplicon sizes of seven candidate reference genes. These genes all passed the specificity and efficiency assessment steps. M: 2k DNA Marker.

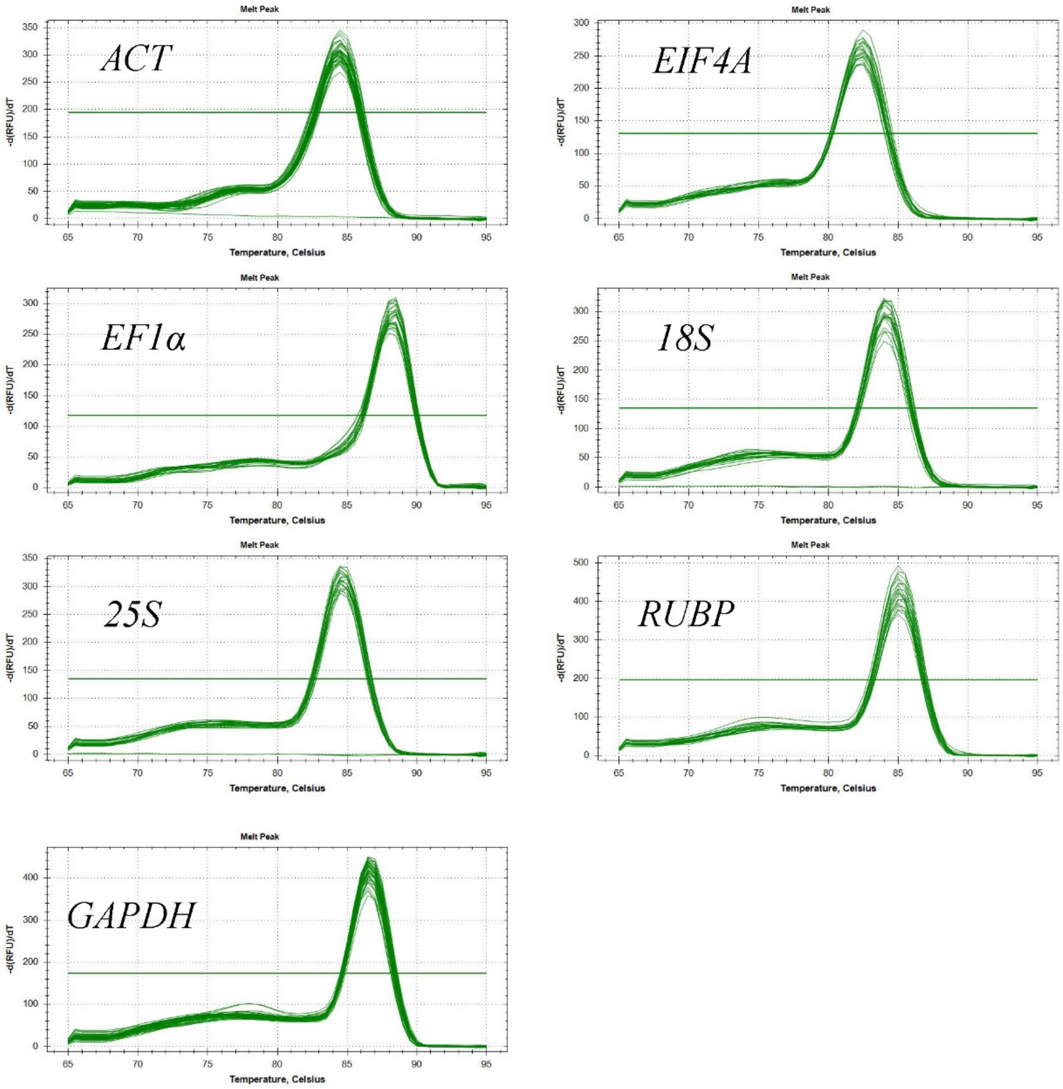

Fig. S2. Evaluation of primer specificity using melting curves.

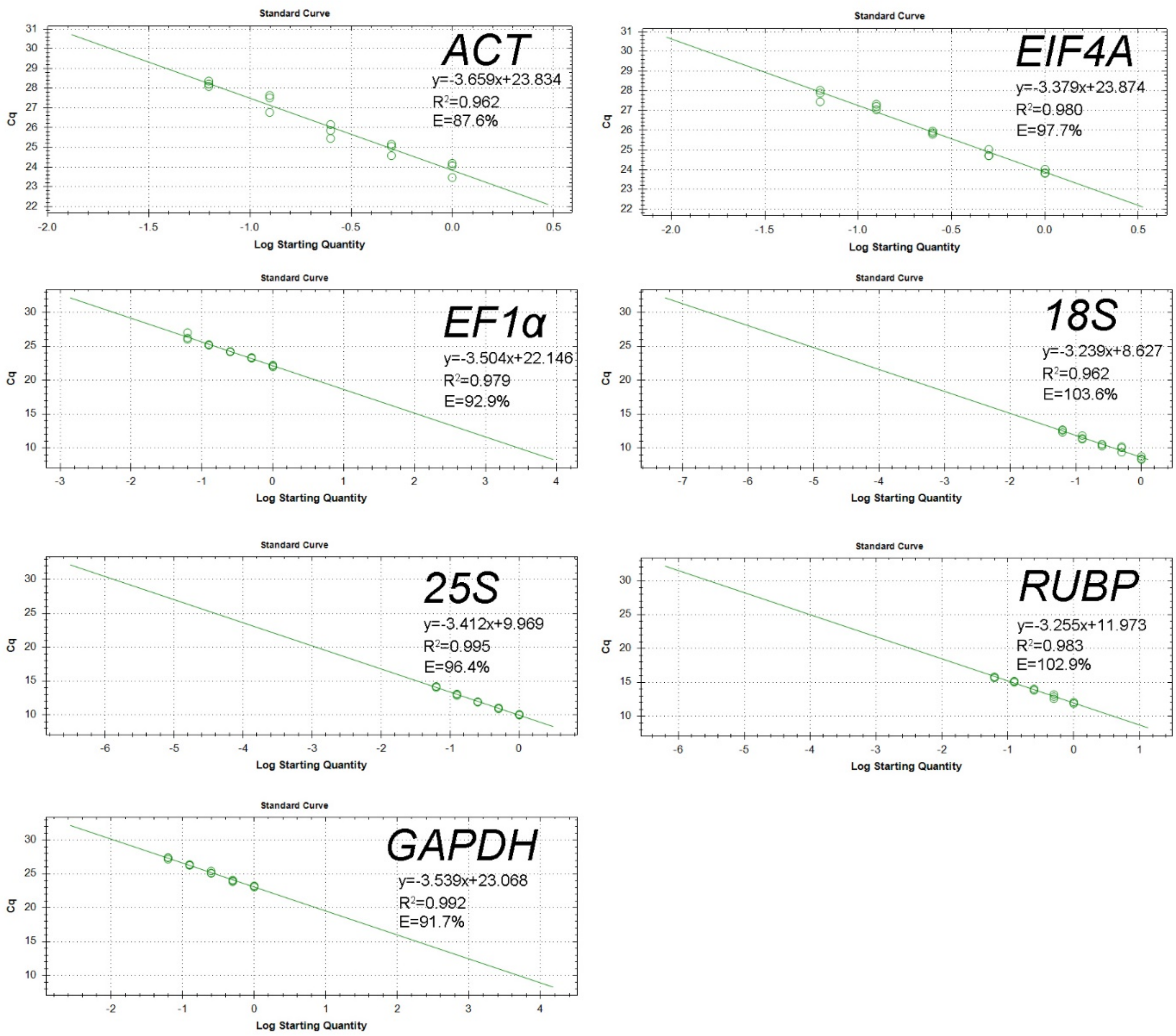

Fig. S3. Standard curves of seven candidate reference genes obtained using RT-qPCR.

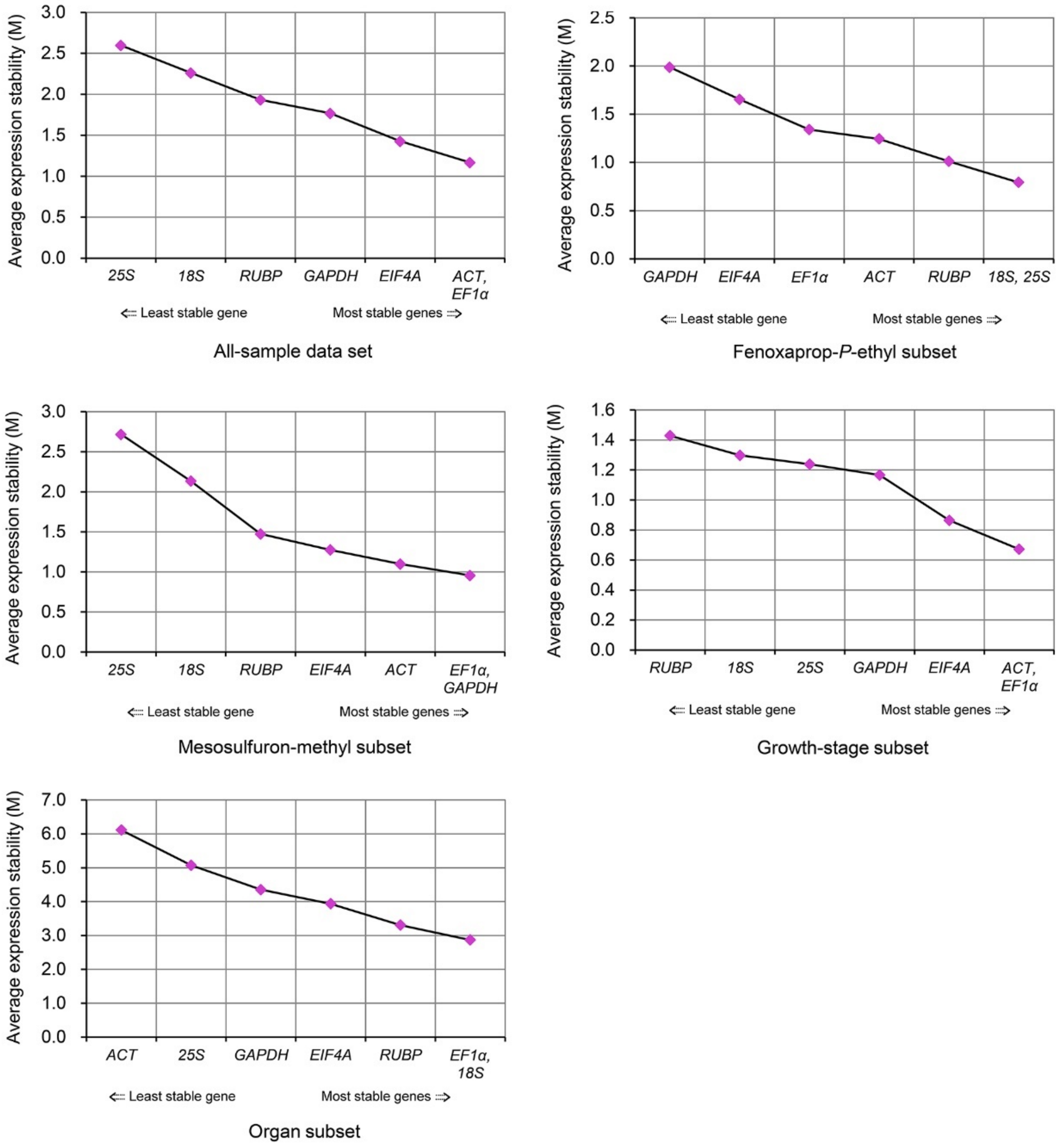

Fig. S4. Expression stability ranking of seven candidate reference genes determined using geNorm. Average expression stability of the reference genes was measured during stepwise exclusion of the least stably expressed reference genes.

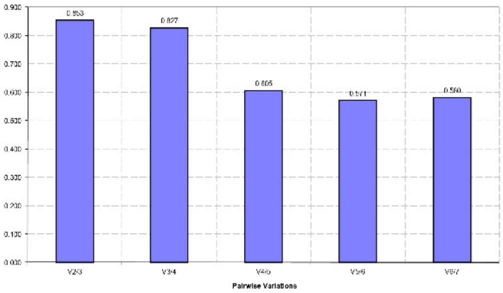

All-samples data set

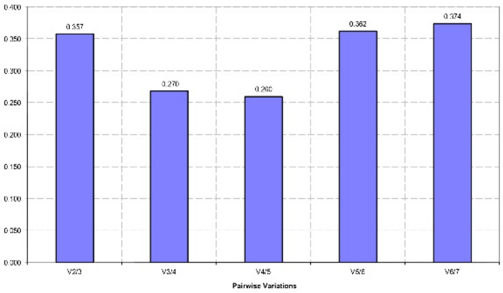

Fenoxaprop-*P*-ethyl subset

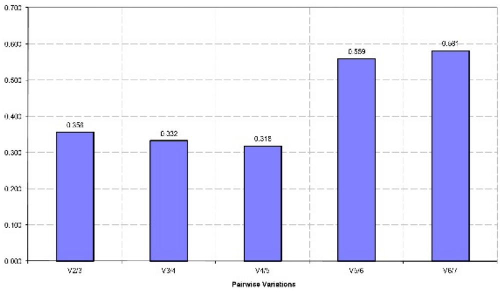

Mesosulfueon-methyl subset

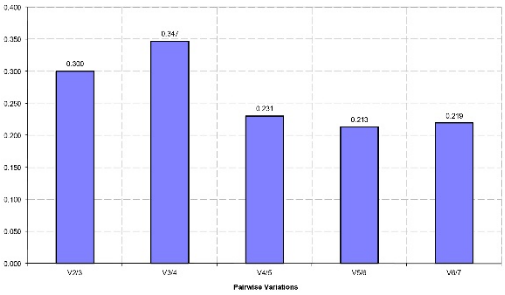

Growth-stage subset

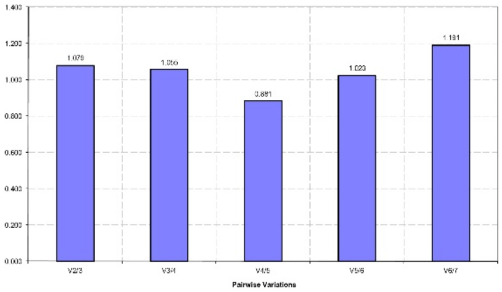

Organ subset

Fig. S5. Pairwise variation (V_n/V_{n+1}) using geNorm to determine the optimal number of reference genes for data normalization in different data (sub)sets.
